# The expression and significance of insulin-like growth factor-1 receptor and its pathway on breast cancer stem/progenitors

**DOI:** 10.1186/bcr3423

**Published:** 2013-05-12

**Authors:** Wen-Wei Chang, Ruey-Jen Lin, John Yu, Wen-Ying Chang, Chiung-Hui Fu, Alan Chuan-Ying Lai, Jyh-Cherng Yu, Alice L Yu

**Affiliations:** 1 The Genomics Research Center, Academia Sinica, 128 Academia Road, Section 2, Nankang District, Taipei City 115, Taiwan; 2 School of Biomedical Sciences, Chung Shan Medical University, 110 Chien-Kuo N. Road, Section 1, Taichung City 402, Taiwan; 3 Department of Medical Research, Chung Shan Medical University Hospital, 110 Chien-Kuo N. Road, Section 1, Taichung City 402, Taiwan; 4 Institute of Cellular and Organismic Biology, Academia Sinica, 128 Academia Road, Section 2, Nankang District, Taipei City 115, Taiwan; 5 Graduate Institute of Life Sciences, National Defense Medical Center, 161 Minquan E. Road, Section 6, Neihu District, Taipei City 114, Taiwan; 6 Taiwan International Graduate Program, Academia Sinica, 128 Academia Road, Section 2, Nankang District, Taipei City 115, Taiwan; 7 General Surgery, Department of Surgery, Tri-Service General Hospital, 161 Minquan E. Road, Section 6, Neihu District, Taipei City 114, Taiwan; 8 Department of Pediatrics, University of California in San Diego, 200 W Arbor Drive, San Diego 92103-8447, USA

## Abstract

**Introduction:**

Dysregulation of the insulin-like growth factor-1 receptor (IGF-1R)/phosphatidylinositol-3-kinase (PI3K)/Akt pathway was shown to correlate with breast cancer disease progression. Cancer stem cells are a subpopulation within cancer cells that participate in tumor initiation, radio/chemoresistance and metastasis. In breast cancer, breast cancer stem cells (BCSCs) were identified as CD24^-^CD44^+ ^cells or cells with high intracellular aldehyde dehydrogenase activity (ALDH^+^). Elucidation of the role of IGF-1R in BCSCs is crucial to the design of breast cancer therapies targeting BCSCs.

**Methods:**

IGF-1R expression in BCSCs and noncancer stem cells sorted from xenografts of human primary breast cancers was examined by fluorescence-activated cell sorting (FACS), western blot analysis and immunoprecipitation. The role of IGF-1R in BCSCs was assessed by IGF-1R blockade with chemical inhibitor and gene silencing. Involvement of PI3K/Akt/mammalian target of rapamycin (mTOR) as the downstream pathway was studied by their phosphorylation status upon IGF-1R inhibition and the effects of chemical inhibitors of these signaling molecules on BCSCs. We also studied 16 clinical specimens of breast cancer for the expression of phosphor-Akt in the BCSCs by FACS.

**Results:**

Expression of phosphorylated IGF-1R was greater in BCSCs than in non-BCSCs from xenografts of human breast cancer, which were supported by western blot and immunoprecipitation experiments. The sorted IGF-1R-expressing cells displayed features of cancer stem/progenitors such as mammosphere formation *in vitro *and tumorigenicity *in vivo*, both of which were suppressed by knockdown of IGF-1R. A specific inhibitor of the IGF-1R, picropodophyllin suppressed phospho-Akt^Ser473 ^and preferentially decreased ALDH^+ ^BCSC populations of human breast cancer cells. Furthermore, picropodophyllin inhibited the capacity of CD24^-^CD44^+ ^BCSCs to undergo the epithelial-mesenchymal transition process with downregulation of mesenchymal markers. Inhibitors of signal molecules downstream of IGF-1R including PI3K/Akt/mTOR also reduced the ALDH^+ ^population of breast cancer cells. Furthermore, the mTOR inhibitor, rapamycin, suppressed BCSCs *in vitro *and *in vivo*.

**Conclusion:**

Our data support the notion that IGF-1R is a marker of stemness, and IGF-1R and its downstream PI3K/Akt/mTOR pathway are attractive targets for therapy directed against breast cancer stem/progenitors.

## Introduction

Cancers are well known to consist of heterogeneous populations of cells that differ in marker expression, proliferation capacity, and tumorigenicity [1,2]. The existence of cancer stem cells (CSCs) has been reported in a variety of malignancies, including leukemia [3], and solid tumors such as brain cancer [4], breast cancer [5], and colon cancer [6]. In breast cancer, CD24^-^CD44^+ ^[5] or cells with high aldehyde dehydrogenase (ALDH) activity [7] have been shown to be enriched in breast cancer stem cells (BCSCs). In addition to their tumor-initiating capacity, BCSCs were reported to be radiation resistant [8] and prone to metastasis [9,10]. Eradication of BCSCs is thus a key to curative therapy of breast cancer [11], and identifying pathways crucial for BCSCs may provide valuable clues for therapeutic targets.

The phosphatidylinositol-3-kinase (PI3K)/Akt (also known as protein kinase B) pathway has been demonstrated to be dysregulated in many types of cancer, including breast cancer [12], and to be associated with poor prognosis [13,14]. In tumors, hyperactivation of the PI3K/Akt pathway may occur by activation of upstream growth factor receptors, overexpression or amplification of Akt, or inactivation of a phosphatase and tensin homolog tumor suppressor [15]. One of the growth receptors associated with activation of Akt is insulin-like growth factor-1 receptor (IGF-1R), which can turn on the signaling cascade of the PI3K/Akt/mammalian target of rapamycin (mTOR) pathway upon stimulation with insulin-like growth factor-1 (IGF-1) [16]. The expression of IGF-1 in breast cancer tissues [17] and serum of breast cancer patients [18] was significantly higher than those in normal healthy individuals. Besides, overexpression and hyperphosphorylation of the IGF-1R in primary breast tumors were reported to correlate with radioresistance and tumor recurrence [19]. Although the IGF-1/IGF-1R pathway seems to be important in breast cancer, its role in BCSCs remains to be delineated. In this study, we investigated the possibility that IGF-1R signal might play an important role in the tumorigenicity and maintenance of BCSCs.

## Methods

### Ethics statement

All of the studies involving human participates were fully encoded to protect patient confidentiality and were utilized under a protocol approved by the Institutional Review Board of Human Subjects Research Ethics Committees of Tri-Service General Hospital and by Academia Sinica, Taipei, Taiwan. All patients enrolled in this study have signed an informed consent form to agree to participate in this study and for publication of the results.

All of the animal studies were operated following a protocol approved by the Institutional Animal Care & Utilization Committee of Academia Sinica, Taipei, Taiwan.

### Isolation and transplantation of primary tumor cells

Primary breast cancer cells were harvested from tumor tissues as described previously [20]. All human breast cancer specimens were obtained from patients who had undergone initial surgery at the Tri-Service General Hospital (Taipei, Taiwan). Samples were fully encoded to protect patient confidentiality and were utilized under a protocol approved by the Institutional Review Board of Human Subjects Research Ethics Committees of Tri-Service General Hospital and Academia Sinica, Taipei, Taiwan.

After receiving the specimens, tumor mass was sliced into 1 mm pieces and digested with collagenase/hyalurondiase digestion buffer (StemCell Technologies, Vancouver, BC, Canada) at 37°C for 2 hours. The released tumor cells were collected after filtration with a 40 μm cell strainer (BD Biosciences, San Jose, CA, USA). Before inoculation of primary tumor cells, 8-week-old female NOD/SCID mice (Tzu Chi University, Hualien, Taiwan) received a sublethal dose of gamma irradiation. For initial establishment and serial passages of xenografts, 1×10^6 ^tumor cells were mixed with 5×10^5 ^normal human breast fibroblasts/site in 2 mg/ml Matrigel and were subcutaneously injected into mammary fat pads of mice. For CSC frequency determination, a serial dilution of sorted tumor cells was mixed with normal human breast fibroblasts and Matrigel and was injected into mammary fat pads of NOD/SCID mice as described above. The tumor formation was monitored weekly. CSC frequency was calculated by Extreme Limiting Dilution Analysis software [21].

### Fluorescence-activated cell sorting

Anti-CD24-PE, anti-CD44-APC, anti-H2K^d^-FITC, and anti-IGF-1R-PE antibody were purchased form BD Biosciences and the ALDEFLUOR assay kit was purchased from StemCell Technologies. Cell labeling with fluorescent-conjugated antibodies or ALDEFLUOR assay was performed according to the manufacturer's recommendations. Sorting of antibody-labeled cells was carried out on a FACSAria cell sorter (BD Biosciences).

### Cell culture and reagents

Sorted H2K^d-^CD24^-^CD44^+ ^cells from BC0145 xenograft and H2K^d-^ALDH^+ ^cells from BC0244 xenograft were cultured in MEM containing 10% fetal bovine serum and insulin (10 μg/ml) at 37°C with 5% CO_2 _and designated AS-B145 and AS-B244, respectively. They could be propagated in serial passages, with emergence of phenotypic diversity of ALDH activity as noted in xenografted tumors. These cultured cells served as convenient *in vitro *cell models for investigating the signaling pathways involved in the maintenance of BCSCs. CB-124005 (Akt inhibitor), PI-103 (PI3K/mTOR inhibitor), rapamycin (mTOR inhibitor), and picropodophyllin (PPP; IGF-1R inhibitor) were purchased from Calbiochem (Billerica, MA, USA), and FPA-124 (Akt inhibitor) was purchased from Tocris Bioscience (Bristol, BS, UK). All of the small-molecule inhibitors were dissolved in dimethylsulfoxide.

### Knockdown of IGF-1R expression

Negative control siRNA or IGF-1R-specific siRNA were purchased from Santa Cruz Biotechnology (Dallas, TX, USA) and delivered into cells by Metafectene SI transfection reagent (Biontex Laboratories GmbH, Martinsried, Germany) at 100 nM according to the manufacturer's protocol. For *in vivo *xenograftment assay, knockdown of IGF-1R was performed by lentivirus-mediated gene silencing. The lentivirus that carry luciferase-specific shRNA (sh-Luc) or IGF-1R-specific shRNA (sh-IGF-1R) were obtained from the National RNAi Core Facility at the Institute of Molecular Biology, (Academia Sinica, Taipei, Taiwan), produced and transduced into cells as described previously [20].

### Fluorescence-activated cell sorting analysis of pAkt^Ser473 ^and E-cadherin

Tumor cells from primary breast tumor tissue were resuspended in staining buffer (0.2% BSA in PBS containing 0.05% NaN_3_) containing an antibody against phosphor-Akt (Ser473; BD Biosciences), anti-CD45-PerCP-Cy5.5, anti-CD24-PE, and anti-CD44-APC. Phosphor-Akt^Ser473^-expressing cells in BCSCs (CD45^-^/CD24^-^/CD44^+^) and non-BCSCs (other cells in the CD45^- ^population) were further analyzed with FACSCalibur (BD Biosciences) flow cytometer and WinMDI software (The Scripps Research Institute, La Jolla, CA, USA). For determination of E-cadherin expression by fluorescence-activated cell sorting (FACS), cells were harvested by 5 mM ethylenediamine tetraacetic acid treatment, incubated with mouse monoclonal anti-E-cadherin antibody (Santa Cruz Biotechnology), followed by Alexa-488 conjugated secondary antibody (Molecular Probes, Grand Island, NY, USA).

### Mammosphere formation assay

Cells were resuspended in Dulbecco's MEM-F12 medium containing 1% methyl cellulose to avoid cell aggregation, and basic fibroblast growth factor (20 ng/ml; PeproTech, Rocky Hill, NJ, USA), human epidermal growth factor (20 ng/ml; PeproTech), insulin (5 μg/ml), and B27 supplement (at a 50× dilution; GIBCO, Grand Island, NY, USA). Cells were seeded at 1,000 cells/well into ultralow-attachment 96-well plates (Corning Life Sciences, Tewksbury, MA, USA). After 7 days of incubation, the number of mammospheres was counted using bright-field optical microscopy under a 20× objective lens, and data were presented as the sphere number per 1,000 cells.

### Western blot analysis

Cells were lysed in RIPA lysis buffer containing NP-40. Twenty-five micrograms of extracted protein was separated using a 4 to 12% gradient NuPAGE (Invitrogen, Grand Island, NY, USA) and transferred to a polyvinylidene difluoride membrane (Immobilon-P; Millipore, Billerica, MA, USA). The membrane was then incubated with antibodies against Akt, phosphor-Akt (Ser473), mTOR, phosphor-mTOR (Ser2448), GAPDH (Cell Signaling Technology, Danvers, MA, USA), phospho-insulin receptor (Tyr972), insulin receptor (IR; GeneTex Inc., Irvine, CA, USA) phospho-IGF-1R (Tyr1165/1166; Santa Cruz Biotechnology), β-actin (Sigma-Aldrich, St. Louis, MO, USA), and the IGF-1R (R&D Systems, Minneapolis, MN, USA). Alkaline phosphatase-conjugated anti-rabbit or anti-mouse immunoglobulin G (Promega, Madison, WI, USA) was used as the secondary antibody. Fluorescent signals from catalyzed ECF substrate were scanned using a Typhoon9400 Variable Mode Imager (Amersham BioScience, Pittsburgh, PA, USA). The quantifications of band intensities were calculated with ImageJ software (National Institutes of Health, Bethesda, MD, USA) or Bio1D (Vilber Lourmat, Marne-la-Vallée, France).

### p-IGF-1R^Tyr1165/1166 ^analysis after immunoprecipitation of IGF-1Rβ

Total cell lysates (500 μg) from sorted ALDH^- ^or ALDH^+ ^BC0244 xenograft tumor cells were used for immunoprecipitation analysis. Briefly, 1 μg IGF-1Rβ specific antibody (sc-713; Santa Cruz Biotechnology) was added into cell lysates (500 μg/200 μl Tris-buffered saline) and incubated at 4°C overnight. After adding 10 μl Protein G Mag Sepharose beads (GE Healthcare Life Science, Pittsburgh, PA, USA), the solutions were further incubated for 2 hours at room temperature. The beads were then proper washed and the binding proteins were eluted by 1× SDS-PAGE sample loading dye. The eluted proteins were further separated by 10% SDS-PAGE and blotted with anti-p-IGF-1R^Tyr1165/1166 ^and anti-IGF-1Rβ antibodies according the protocol of western blot analysis.

### Cell migration assay

Cells were suspended in serum-free culture medium, seeded at in the upper chamber insert of a transwell plate (Corning Life Sciences) and then inserted into 24-well plates with serum-containing medium. After incubation at 37°C for 16 hours, cells that had migrated across the membrane of the insert were stained with crystal violet after removing the cells attached on the inner face of the insert and results were recorded by microscopy.

### Immunofluorescence staining of E-cadherin

Cells were fixed with cold methanol followed by 3.7% formaldehyde/PBS. After blocking with 1% BSA/PBS, cells were incubated with an anti-E-cadherin antibody and then further incubated with an Alexa-488-conjugated secondary antibody. Fluorescence signals were captured under an inverted fluorescence microscope (Olympus, Shinjuku-ku, Tokyo, Japan).

## Results

### Increased IGF-1R activity in BCSCs of xenograft of human breast cancer

Xenografts of two human breast cancers, BC0145 and BC0244, were established by inoculating primary human breast cancer cells in the mammary fat pads of NOD/SCID mice. BC0145 tumor was estrogen receptor (ER)-negative, progesterone receptor (PR)-positive, HER2/neu-positive, and BC0244 was triple negative. The engrafted tumors displayed similar histology and expression status of ER/PR/Her2 as the patients' specimens [20] (see Figure S1 in Additional file 1). To determine the BCSC population in BC0145 and BC0244 xenografts, CD24^-^CD44^+ ^and ALDH+ cells were sorted from H2K^d- ^cells using FACS (see Figure S2 in Additional file 1) and injected into the mammary fat pads of NOD/SCID mice. The xenograftment results indicated that CSCs could be enriched in H2K^d-^CD24^-^CD44^+ ^or H2K^d-^ALDH^+ ^cells because of their higher tumorigenicity (see Table S1 in Additional file 1) and *in vivo *re-emergence of heterogeneity as their parental tumors (see Figure S2D for CD24^-^CD44^+ ^and Figure S2G,H for ALDH^+ ^in Additional file 1). These two xenografted human breast cancers are suitable for investigating the characteristics of BCSCs.

**Figure 1:**
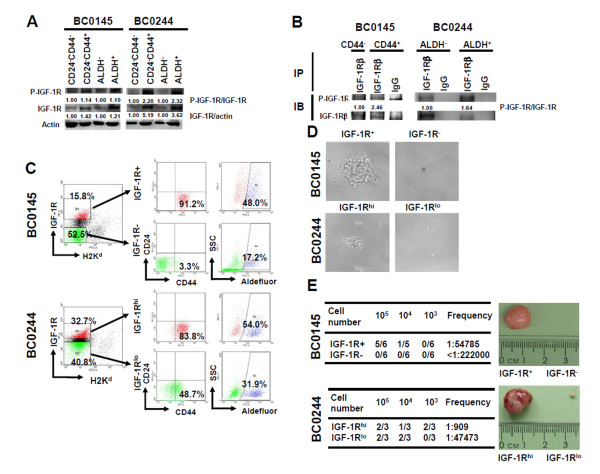
**Insulin-like growth factor 1 receptor serves as a marker for breast cancer stem cells**. **(A) **Cells from xenograft BC0145 or BC0244 tumors were sorted as indicated populations and phosphorylation of insulin-like growth factor 1 receptor (IGF-1R) was determined by western blot. **(B) **pIGF-1R^Tyr1165/1166 ^of immunoprecipitated IGF-1R from aldehyde dehydrogenase (ALDH)^- ^or ALDH^+ ^BC0244 xenograft tumor cells was determined. **(C) **Tumor cells of BC0145 or BC0244 xenografts were stained with PE-conjugated anti-IGF-1R antibody and FITC-conjugated anti-H2K^d ^antibody. CD24^-^CD44^+ ^or ALDH^+ ^cells within IGF-1R^+^/IGF-1R^- ^BC0145 cells (upper panel in (B)) or IGF-1R^hi^/IGF-1R^lo ^BC0244 cells (lower panel in (B)) were determined by co-stain with PE-Cy7-conjugated anti-CD24/APC-conjugated anti-CD44 antibodies or Aldefluor substrate. **(D), (E) **Two populations of IGF-1R^+^/IGF-1R^- ^(BC0145) or IGF-1R^hi^/IGF-1R^lo ^(BC0244) cells were sorted from the H2K^d- ^population by fluorescence-activated cell sorting (FACS) and determined the mammosphere formation capability (D) or tumorigenicity (E). The CSC frequency was calculated by ELDA software (table in (E)). All experiments were repeated independently at least twice and results shown were from a representative experiment. IP, immunoprecipitation; IB, immunoblot.

We next compared the activation status of the IGF-1R in BCSCs and non-BCSCs sorted from BC0145 and BC0244 xenografts by western blot. The amount of the phosphorylated IGF-1R^Tyr1165/1166 ^was greater by 1.10-fold to 2.32-fold in CD24^-^CD44^+ ^and ALDH^+ ^BCSCs than non-CD24^-^CD44^+ ^and ALDH^- ^cells in both xenografts (Figure 1A). The total IGF-1R in the BCSC-enriched population was also 1.23-fold to 5.19-fold that of non-BCSCs. We further performed chromatin immunoprecipitation analysis to support the western blot results of p-IGF-1RTyr1165/1166 because of the cross-reactivity between p-IGF-1R and phosphorylated IR. After immunoprecipitation with IGF-1Rβ-specific antibody, p-IGF-1RTyr1165/1166 was also increased 1.64-fold in ALDH^+ ^BC0244 xenograft tumor cells when compared with ALDH^- ^cells (Figure 1B). In line with these findings, the levels of *IGF1R *mRNAs were also increased in CD24^-^CD44^+ ^BC0145 and ALDH^+ ^BC0244 BCSCs (see Figure S3A in Additional file 1). To distinguish the possible involvement of IR, we also examined the expression of IR and phosphorylated IR in BCSCs and non-BCSCs. Unexpectedly, the IR expression as well as its phosphorylation in BCSCs of BC0145 xenograft cells was markedly lower than those in non-BCSCs, but there was no obvious difference between BCSCs and non-BCSCs of BC0244 xenograft cells (see Figure S3B in Additional file 1). These findings suggest that IGF-1R, but not IR, is activated to a greater extent in BCSCs than non-BCSCs and that IGF-1R signaling may play a crucial role in BCSCs.

### IGF-1R serves as a novel marker for breast cancer stem/progenitors

Given the importance of IGF-1R signaling in the progression of breast cancer, we next examined whether IGF-1R could serve as a marker for BCSCs. FACS analysis of BC0145 revealed that 91.2% and 48.0% of IGF1R^+ ^cells were CD24^-^CD44^+^, and ALDH^+^, respectively, as compared with 3.3% and 17.2% of IGF1R^- ^cells bearing these markers, respectively (Figure 1C, upper panel). As for BC0244 xenograft tumor cells, 83.8% and 54% of IGF1R^hi ^were CD24^--^CD44^+^and ALDH^+^, respectively, as compared with 48.7% and 31.9% of IGF-1R^lo ^cells, respectively (Figure 1C, lower panel). These results indicated that IGF-1R^hi ^breast cancer cells were enriched for CSC markers. We next used western blot to confirm the expression of IGF-1R in IGF-1R^+^/IGF-1R^hi ^cells and the results showed greater expression of total IGF-1R protein level in sorted IGF-1R^+^/IGF-1R^hi ^cells (2.66-fold or 1.25-fold in IGF-1R^+ ^BC0145 cells or IGF-1R^hi ^BC0244 cells, respectively) as well as total P-IGF-1R (2.05-fold or 1.24-fold in IGF-1R^+ ^BC0145 cells or IGF-1R^hi ^BC0244 cells, respectively), although the ratio of P-IGF-1R/IGF-1R was less in IGF-1R^+ ^BC0145 cells (see Figure S3C in Additional file 1).

**Figure 2:**
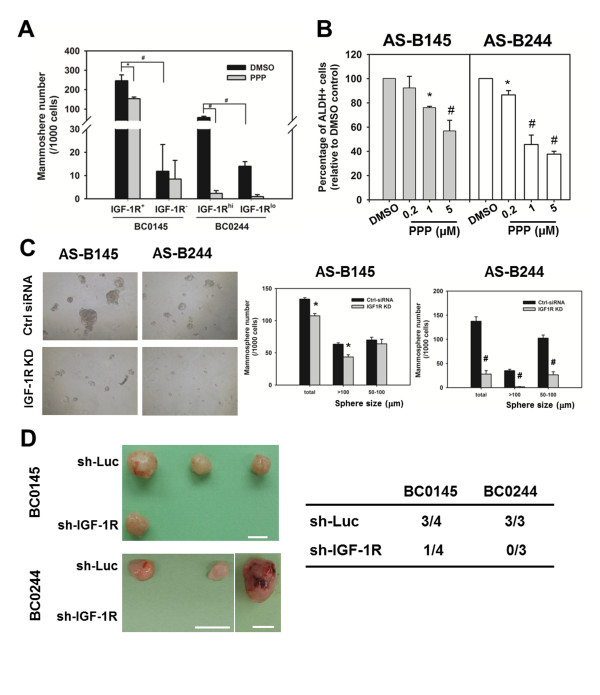
**Disruption of insulin-like growth factor 1 receptor activity diminished the breast cancer stem cell population**. **(A) **Two populations of insulin-like growth factor 1 receptor (IGF-1R)^+^/IGF-1R^- ^(BC0145) or IGF-1R^hi^/IGF-1R^lo ^(BC0244) cells were sorted as described in Figure 1B and the mammosphere formation capability determined in the presence of 0.1% dimethylsulfoxide (DMSO) or 1 μM picropodophyllin (PPP). *P <0.05; ^#^*P *<0.01. **(B) **The mammosphere forming capability of AS-B145 or AS-B244 cells under PPP or 0.1% DMSO treatment was determined. *P <0.05; ^#^*P *<0.01. **(C) **Cells were transfected with 100 nM of negative control siRNA (ctrl siRNA) or IGF-1R specific siRNA (IGF-1R KD) and cells were harvested to determine the mammosphere forming capability. *P <0.05; ^#^*P *<0.01. **(D) **IGF-1R^+ ^BC0145 or IGF-1R^hi ^BC0244 cells were sorted, transduced with sh-Luc or sh-IGF-1R lentivirus and the tumorigenicity determined in NOD/SCID mice. All experiments were repeated independently at least twice and results shown were from a representative experiment. ALDH, aldehyde dehydrogenase.

To confirm whether IGF-1R could serve as a marker for BCSCs, BC0145 or BC0244 xenograft tumor cells were sorted into IGF-1R^+^/IGF-1R^- ^(BC0145) or IGF-1R^hi^/IGF-1R^lo ^cells (BC0244), respectively (the purity of FACS are shown in Figure S3D in Additional file 1), and tested for tumorigenicity *in vitro *and *in vivo*. As expected, IGF-1R^+ ^BC0145 or IGF-1R^hi ^BC0244 cells displayed greater capacity of mammosphere formation, with increased size (Figure 1D) and number (Figure 2A) of spheres. *In vivo*, IGF-1R^+ ^BC0145 and IGF-1R^hi ^BC0244 cells displayed greater tumorigenicity in NOD/SCID mice than IGF-1R^- ^BC0145 and IGF-1R^lo ^BC0244 cells (Figure 1E). For BC0145, the CSC frequency of IGF-1R^+ ^cells was higher than IGF-1R^- ^cells, which failed to generate any tumor with up to 10^5 ^cells (*P *= 0.0036). Similar results were observed in BC0244 (*P *= 8.94×10^-78^). In addition, tumor cells derived from IGF-1R^+ ^BC0145 cells or IGF-1R^hi ^BC0244 cells displayed phenotypic diversity in IGF-1R expression as the original tumor (see Figure S4A in Additional file 1). Further analysis of these tumors showed that more than 90% of IGF-1R^hi ^cells were CD24^-^CD44^+ ^but less than 30% of IGF-1R^-/lo ^were CD24^-^CD44^+ ^(see Figure S4B in Additional file 1), indicating the capacity of IGF-1R^+^/IGF-1R^hi ^cells to undergo differentiation when they formed tumors *in vivo*.

### IGF-1R blockade abolishes the cancer stem/progenitor features *in vitro *and *in vivo*

To further support the use of IGR-1R as a marker for breast cancer stem/progenitors, the effects of IGF-1R inhibition on the CSC features were determined. Upon treatment with picropodophyllin (PPP), a specific small-molecule inhibitor of the IGF-1R that has no effects on the related receptor tyrosine kinases such as insulin receptor [22] and epidermal growth factor receptor [23], the mammosphere forming capacity of IGF-1R^+ ^BC0145 and IGF-1R^hi ^BC0244 cells was significantly reduced (Figure 2A).

To facilitate further studies of the role of IGF-1R, we established cultured cell lines derived from H2K^d-^CD24^-^CD44^+ ^and H2K^d-^ALDH^+ ^cells of xenografts of BC0145 and BC0244, respectively. These cells could be propagated in serial passages with emergence of phenotypic diversity of ALDH activity as noted in parental tumors. These cultured cells derived from BCSCs of BC0145 and BC0244 were designated AS-B145 and AS-B244, respectively, and served as convenient *in vitro *cell models for investigating the signaling pathways involved in the maintenance of BCSCs. Incubation of AS-B145 and AS-B244 cells with PPP for 48 hours resulted in a dose-dependent decrease in their ALDH^+ ^population (Figure 2B). Furthermore, silencing of IGF-1R in AS-B145 cells inhibited the total number of mammospheres to 80.5 ± 2.6% of the control siRNA (*P *= 0.012) and 64.4 ± 4.4% (*P *= 0.021) for those spheres >100 μm. The suppressive effect was even more pronounced for AS-B244 cells, down to 25.9 ± 6.2% (*P *= 0.007) and 4.3 ± 2.0% (*P *= 0.004) for the total number of mammosphere and larger spheres, respectively (Figure 2C).

We next determined whether knockdown of IGF-1R suppresses the tumorigenicity of IGF-1R expressing breast cancer stem/progenitors. Lentivirus-mediated silencing of IGF-1R in IGF-1R^+ ^BC0145 or IGF-1R^hi ^BC0244 cells suppressed their tumorigenicity in NOD/SCID mice, with tumor formation in only one out of four mice by week 9 after injection of sh-IGF-1R transduced IGF-1R^+ ^BC0145 cells, and no tumor formation up to week 15 after injection of sh-IGF-1R transduced IGF-1R^hi ^BC0244 cells. In contrast, tumor formation was noted in three of four mice at week 9 or three of three mice by week 15 after injection of sh-Luc transduced IGF-1R^+ ^BC0145 or IGF-1R^hi ^BC0244 cells, respectively (Figure 2D; see also Figure S4C in Additional file 1). Interestingly, the percentage of IGF-1R^+ ^cells (4.3%) in the single tumor derived from the sh-IGF-1R group was significantly less than that from the sh-Luc group (35.2%), whereas the percentage of CD24^-^CD44^+ ^cells was similar between the two groups (34.9% and 37.2% in the sh-IGF-1R group and sh-Luc group, respectively) (see Figure S4D in Additional file 1). Taken together, IGF-1R inhibition not only decreased the CSC population of breast cancer but also suppressed the mammosphere formation and tumor growth of IGF-1R^+ ^cells. These results lent further support that IGF-1R could serve as a novel marker for breast cancer stem/progenitors and that IGF-1R signaling was crucial in the maintenance of this particular population within breast cancer.

**Figure 3:**
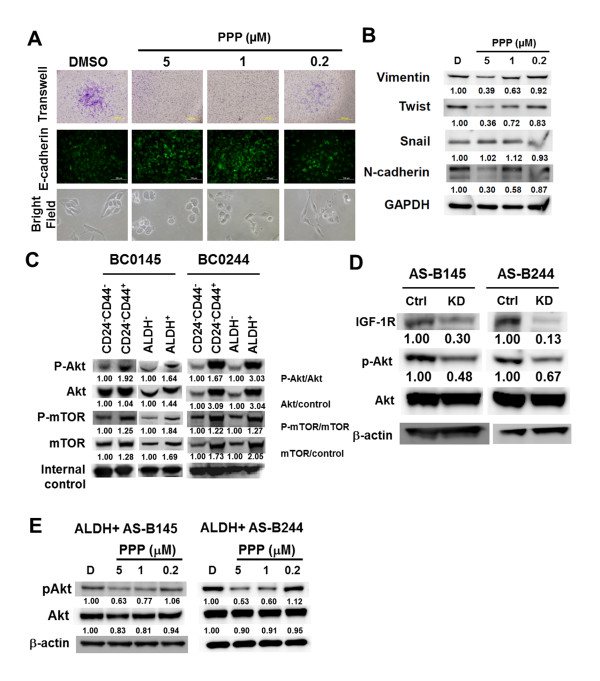
**Targeting IGF-1R affects cancer stem cell properties and Akt activation of breast cancer stem cells**. **(A) **CD44^+ ^cells of AS-B244 cells were treated with picropodophyllin (PPP) for 12 hours and cell migration within 18 hours was determined (upper panel). E-cadherin expression was determined by immunofluorescence stain (middle panel, 10× objective lens). Cell morphology was observed under microscope (bright field, 20× objective lens). **(B) **The expression of epithelial-mesenchymal transition-related molecules after PPP treatment was determined by western blot. **(C) **Cells were sorted as described in Figure 1A and the phosphorylation of Akt or mammalian target of rapamycin (mTOR) was determined by western blot. GAPDH and actin were used as internal control for BC0145 and for BC0244, respectively. **(D) **AS-B145 or AS-B244 cells were transfected with 100 nM negative control siRNA (ctrl) or insulin-like growth factor 1 receptor (IGF-1R) specific siRNA (KD) for 48 hours and the expression of IGF-1R, Akt or p-Akt was determined by western blot. **(E) **ALDH^+ ^AS-B145 or ALDH^+ ^AS-B244 cells were treated with PPP for 48 hours. pAkt^ser473^was determined by western blot. All experiments were repeated at least twice and results shown were from a representative experiment. ALDH, aldehyde dehydrogenase; DMSO, dimethylsulfoxide.

In view of the reported involvement of IGF-1R signaling in the metastasis [24,25] and epithelial-mesenchymal transition (EMT) [26] of breast cancer cells, we further investigated whether IGF-1R signal also regulates EMT process in CD44^+ ^BCSCs. Incubation of sorted CD44^+ ^AS-B244 cells with PPP suppressed the migration ability of BCSCs in a transwell assay in a dose-dependent manner, with negligible migration at 5 μM (Figure 3A). This was accompanied by a concentration-dependent change of cell morphology from mesenchymal appearance to cuboidal shape (Figure 3A, bright field), although some morphological heterogeneity was noted in CD44^+ ^AS-B244 cells without PPP treatment. Repression of E-cadherin, a hallmark of EMT, was also examined by immunofluorescence staining, which revealed a progressive increase in E-cadherin expression in CD44^+ ^AS-B244 cells incubated with increasing concentrations of PPP (Figure 3A; see also Figure S4E in Additional file 1). Upregulation of E-cadherin by PPP was also confirmed by FACS analysis, with an increase in the percentage and mean fluorescence intensity of E-cadherin-positive cells of sorted CD44^+ ^AS-B244 cells upon treatment with PPP (see Figure S4F in Additional file 1). Analysis of other EMT markers by western blot revealed that PPP treatment led to concentration-dependent decreases in the expression of vimentin, N-cadherin, and twist, but not snail (Figure 3B). These results indicate that IGF-1R signaling is required not only for the survival of BCSCs, but also for their capability to undergo EMT.

### IGF-1R signaling leads to Akt activation in BCSCs

**Figure 4:**
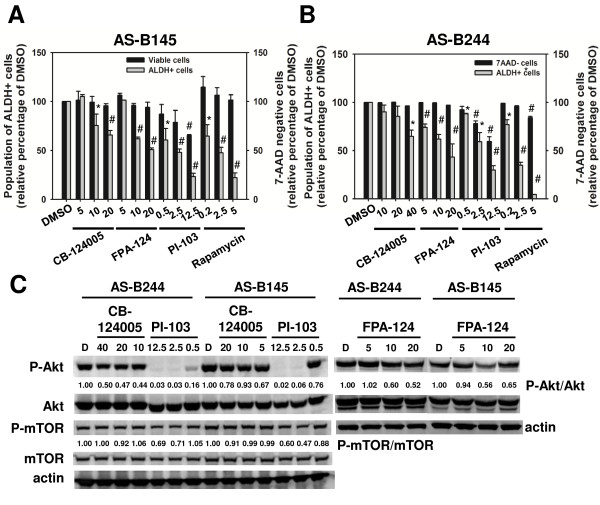
**Targeting the PI3K/Akt/mTOR pathway inhibits the cancer stem cell population of breast cancer cells**. **(A) **AS-B145 cells or **(B) **AS-B244 cells were treated with small molecule inhibitors of phosphoinositide-3-kinase (PI3K)/mammalian target of rapamycin (mTOR) (PI-103), Akt (CB-124005 or FPA-124) or mTOR (rapamycin) for 48 hours and the aldehyde dehydrogenase (ALDH)^+ ^cell population within 7-aminoactinomycin D (7-AAD)-negative cells was determined with Aldefluor assay and displayed as the relative percentage of dimethylsulfoxide (DMSO) control. *P <0.05; ^#^*P *<0.01 when compared with the DMSO group. **(C) **pAkt^ser473 ^or p-mTOR^Ser2448 ^was determined with western blot after treatment with inhibitors for 18 hours. Data are presented as the fold of changes relative to the DMSO control. All experiments were repeated independently at least twice and results shown were collected from a representative experiment.

To examine whether IGF-1R is a possible upstream stimulus of the PI3K/Akt/mTOR pathway in BCSCs, we first tested the activation status of Akt/mTOR between BCSCs and non-BCSCs. The phosphorylation of Akt^Ser473 ^(pAkt^Ser473^) and mTOR^Ser2448 ^(p-mTOR^Ser2448^) was higher in CD24^-^CD44^+ ^and ALDH^+ ^BCSCs; and an increased amount of Akt and mTOR proteins was also noted in ALDH^+ ^BC0145 cells, CD24^-^CD44^+ ^BC0244 cells, and ALDH^+ ^BC0244 cells (Figure 3C). The increased protein level of Akt or mTOR was accompanied by the upregulation of their mRNA (see Figure S4 in Additional file 1). We next tested whether disruption of the IGF-1R signaling will reduce Akt activation. Knockdown of IGF-1R decreased the phosphorylation of Akt^Ser473 ^in both AS-B145 and AS-B244 cells (Figure 3D). Similar results were observed in PPP-treated ALDH^+ ^AS-B145 and ALDH^+ ^AS-B244 cells in a dose-dependent manner at 18 hours (Figure 3E). We further examined the effects of disruption of the PI3K/Akt/mTOR pathway in the CSC population of breast cancer cells. AS-B145 and AS-B244 cells were incubated with a PI3K/mTOR inhibitor (PI-103), Akt specific inhibitors (CB-124005 and FPA-124), or rapamycin for 48 hours, and the number of viable BCSC population identified as ALDH^+ ^7-AAD^- ^cells was determined by FACS analysis. The percentages of ALDH^+ ^cells in both AS-B145 (Figure 4A) and AS-B244 cells (Figure 4B) diminished upon treatment with PI-103, CB-124005, FPA-124, and rapamycin in dose-dependent manners, with more pronounced inhibition by PI-103 and rapamycin. This was accompanied by a decrease in Akt^Ser473 ^phosphorylation after treatment with PI-103, CB-124005, and FPA-124, although the phosphorylation of mTOR, the downstream signal molecule of Akt, was suppressed only by PI-103 (Figure 4C).

**Figure 5:**
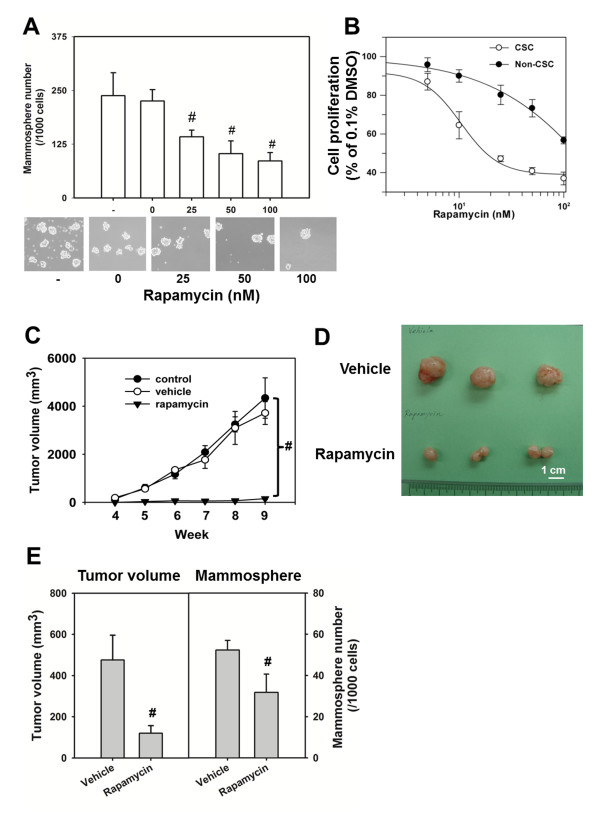
**Rapamycin suppresses stem cell population, *in vitro *cell proliferation and *in vivo *tumorigenicity**. **(A) **CD24^-^CD44^+ ^cells of BC0145 xenograft tumors were cultured for mammosphere formation for 7 days, and the number of spheres were counted and shown as the number per 1,000 cells. ^#^*P *<0.01. **(B) **Differential sensitivity of breast cancer stem cells (BCSCs) and non-BCSCs to rapamycin *in vitro*. BCSCs and non-BCSCs were cultured at 1×10^4 ^cells/well with rapamycin and cell titers were determined at 48 hours by alamar blue assay. **(C) **Effects of rapamycin on tumorigenicity of BCSCs *in vivo*. Groups of three NOD/SCID mice were inoculated with 10^5 ^cells of BCSCs in the mammary fat pads and treated with intraperitoneal injections of rapamycin every 2 days at 4 mg/kg for 3 weeks. Tumor volumes were monitored weekly from weeks 4 to 9. ^#^*P *<0.01. **(D) **The CD24^-^CD44^+ ^BC0145 cells were injected into mammary fat pads. Two weeks later, the mice were treated with rapamycin at 2 mg/kg/every 2 days for first week and 1 mg/kg/every 2 days for a further 2 weeks. The therapeutic effect of rapamycin was demonstrated by the reduced tumor formation and tumor volume. The BCSC activity in vehicle-treated or rapamycin-treated tumors was determined by mammosphere forming capability. To avoid the influence of mouse cells, the assay was conducted with H2K^d- ^tumor cells. ^#^*P *<0.01. All experiments were repeated independently at least twice and results shown were collected from a representative experiment. CSC, cancer stem cell; DMSO, dimethylsulfoxide.

We next examined whether rapamycin could inhibit the self-renewal of BCSCs *in vitro*. The mammosphere formation capacity of BC0145 xenograft tumor was inhibited by rapamycin in a concentration-dependent manner at 25 to100 nM (Figure 5A). The survival BCSCs were also much more susceptible than non-BCSCs to the inhibitory effects of rapamycin, with IC_50 _of 10.4 ± 1.4 nM and 320.6 ± 99.4 nM for BCSCs and non-BCSCs, respectively (Figure 5B). Furthermore, treatment of NOD/SCID mice with rapamycin for 3 weeks suppressed the *in vivo *tumorigenicity of BCSCs by more than 99% of the vehicle control (Figure 5C). In order to investigate whether rapamycin treatment reduces proliferation of BCSCs *in vivo*, we inoculated 10^5 ^CD24^-^CD44^+ ^cells sorted from BC0145 xenograft. Two weeks later, mice were treated with rapamycin for 3 weeks and the resulting tumors were measured and harvested for assessment of BCSC activity by mammosphere formation assay. As shown in Figure 5D,E, rapamycin inhibited tumorigenicity of BCSCs as reflected by reduced tumor volume to 23.5 ± 7.7% of vehicle control (*P = *0.008) and mammosphere forming capacity of H2K^d- ^tumor cells to 60.8 ± 16.9% of control *(P *= 0.0043). These findings were in sharp contrast to the increased proportion of BCSCs and enhanced mammosphere forming capacity observed after treatment with doxorubicin [27] or taxol [28], lending further support that rapamycin could suppress the survival of BCSCs. These results indicate that the efficient suppression of the PI3K/mTOR pathway by small-molecule inhibitors preferentially purges the BCSC population.

We next examined the activation status of Akt in 16 primary breast cancer specimens. The clinical and histopathological characteristics of these 16 breast cancer patients are summarized in Table S2 of Additional file 1. Freshly harvested tumor cells with CD45^-^CD24^-^CD44^+ ^marker were delineated as BCSCs, with the remaining CD45^- ^population as non-BCSCs, and their expression of intracellular pAkt^Ser473 ^was determined by FACS (see Figure S4A in Additional file 1 for an illustrated an example of BC0417). Among 16 primary human breast cancer specimens, pAkt^Ser473 ^was detected in 11 samples that displayed a significantly higher percentage of pAkt^Ser473^-positive cells in the BCSC population (ranging from 23.3 to 57.8%) than non-BCSCs (0 to 56.2%) (*P *= 0.048) (Table 1). Among these 11 samples with positive pAkt^ser473^, the expression levels of pAkts^er473 ^were higher in BCSCs than non-BCSCs in seven samples, equivalent in two samples, and lower in BCSCs in the remaining two samples (Table 1). There was no obvious correlation between the Akt activation in these 16 patients and their clinical stage (*P *= 0.11) or status of estrogen receptor, progesterone receptor or HER2/neu (*P *= 0.59, 0.83 and 0.30, respectively) (see Table S3in Additional file 1). Combining our previous data [20], we investigated whether there was any correlation between CD24^-^CD44^+ ^percentage and breast cancer subtypes, according to their expression profiles of ER, PR and HER2/neu [29,30]. Among luminal A (ER/PR^+^, HER2/neu^-^), luminal B (ER/PR^+^, HER2/neu^+^), HER2 overexpression (ER^-^, PR^-^, HER2/neu^+^) and triple negative subtypes of breast cancer, the CD24^-^CD44^+ ^percentage was only significantly increased in triple negative breast cancer when compared with luminal B (*P *= 0.0464; see Figure S5B in Additional file 1). Overall, these findings revealed that Akt activation was greater in BCSCs than in non-BCSCs for those samples with detectable p-Akt.

**Table 1:** Phosphorylated Akt^Ser473 ^expression between BCSCs and non-BCSCs from primary human breast cancer specimens

	Expression level of p-Akt^Ser473 ^(%, MFI)^a^		
	
	BCSCs (CD45^-^CD24^-^CD44^+^)	Non-BCSCs (other populations of CD45^- ^cells)	Ratio^b ^(BCSCs/non-BCSCs)
BC0414	33.3 (9.7)	17.5 (9.4)	1.90
BC0417	41.8 (32.0)	29.0 (33.0)	1.44
BC0422	32.4 (16.9)	14.3 (25.2)	2.27
BC0426	23.3 (5.2)	22.6 (5.7)	1.03
BC0450	41.3 (10.3)	56.2 (13.9)	0.73
BC0466	56.7 (36.5)	40.8 (44.1)	1.39
BC0480	55.7 (11.8)	37.8 (9.6)	1.47
BC0485	33.0 (44.2)	43.4 (54.8)	0.76
BC0512	57.8 (66.7)	37.8 (38.2)	1.53
BC0526	53.9 (28.2)	50.8 (34.4)	1.06
BC0533	47.8 (33.8)	0	∞

BSCS, breast cancer stem cell; MFI, mean fluorescence intensity ^a^p-Akt^Ser473 ^expression was determined by flow cytometry as described in Materials and methods. ^b^The ratio was calculated according to the positive percentage of pAkt^Ser473 ^in BCSCs and non-BCSCs.

### IGF-1R participates in the maintenance of BCSCs and Akt activation in ER-positive breast cancer

**Figure 6:**
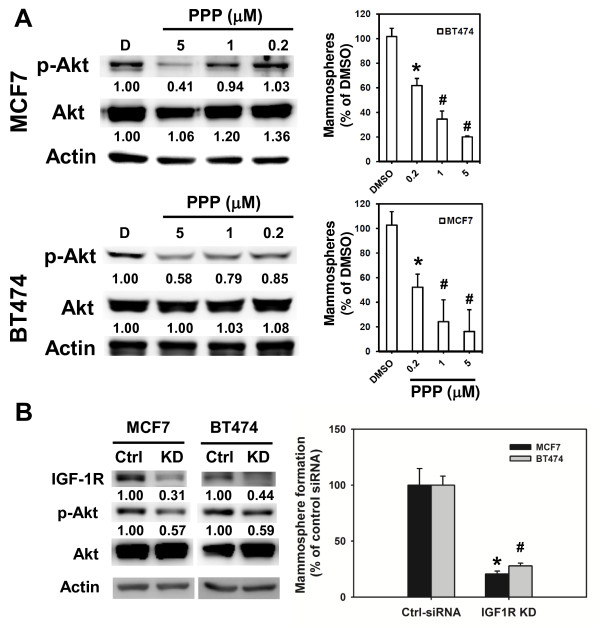
**IGF-1R is essential for the maintenance and Akt activation of estrogen receptor-positive breast cancer cells**. **(A) **MCF7 or BT474 breast cancer cells were treated with picropodophyllin (PPP) and harvested to detect Akt phosphorylation with western blot (right panel) or mammosphere formation capability (left panel). Numbers in the left panel indicated relative expression in comparison with dimethylsulfoxide (DMSO)-treated cells. Data of mammosphere formation assay presented as the relative percentage of DMSO-treated cells. *P <0.05; ^#^*P *<0.01. **(B) **MCF7 or BT474 cells were transfected with negative control siRNA (Ctrl-siRNA) or insulin-like growth factor 1 receptor (IGF-1R) specific siRNA [53] for 48 hours and cells were harvested for detection of Akt phosphorylation by western blot (left panel) or mammosphere formation capability. Data presented as relative percentage of Ctrl-siRNA transfected cells. *P <0.05; ^#^*P *<0.01. All experiments were repeated independently at least twice and results shown were collected from a representative experiment.

The IGF-1R/ insulin receptor substrate-1 pathway is reported to be activated greatly in ER-positive breast cancer cells and contributes to their proliferation and survival. We therefore investigated whether IGF-1R signaling also controls the self-renewal capacity of ER-positive breast cancer cells. Treatment of two ER-positive MCF7 and BT474 breast cancer cell lines with 5 μM PPP significantly inhibited phosphorylation of Akt. Their mammosphere forming capacities were also significantly suppressed by 0.2, 1 or 5 μM PPP in a concentration-dependent manner (Figure 6A). In addition, knockdown of IGF-1R by siRNA also reduced phosphorylated Akt and inhibited mammosphere formation to 27.8 ± 2.4% or 20.5 ± 2.6% of negative control siRNA in BT474 or MCF7 cells, respectively (Figure 6B). These results indicated that the IGF-1R signaling pathway also plays an important role in the maintenance of BCSCs in both ER-positive and ER-negative breast cancers.

## Discussion

In this study, we used the reported BCSC markers, CD44/CD24, and ALDH activity to examine the role of IGF-1R in BCSCs. We showed greater phosphorylation of IGF-1R in BCSCs than in non-BCSCs and preferential sensitivity of BCSCs to PPP, a specific inhibitor of the IGF-1R, leading to reduced phosphorylation of Akt^Ser473 ^and decreased ALDH^+ ^BCSC populations. In human malignancies, increased circulating IGF-1 was associated with a greater risk of several cancers, including breast cancer [18]. A crosstalk between IGF-1R and the Wnt pathway has been reported in colon cancer [31], oligodendroglial cells [32], and chondrocytes [33]. The interaction of these two pathways in breast cancer is intriguing and awaits further investigation. Recently, two reports demonstrated the essential role of IGF/IGF-1R signaling in the maintenance of leukemia-initiating cells in T-cell acute lymphoblastic leukemia [34] or in the transformation of hematopoietic progenitor cells in the mouse model of acute myelogenous leukemia [35]. The IGF-1R expression of leukemia-initiating cells in T-cell acute lymphoblastic leukemia was maintained by Notch signaling [34], which also contributed to the maintenance of BCSCs [36,37]. Whether the Notch pathway is involved in the IGF-1R signaling in BCSCs remains to be investigated. In solid tumors, chemoresistant colorectal cells displayed a CSC phenotype and became more sensitive to IGF-1R inhibition [38]. In hepatocellular carcinoma, the IGF-2/IGF-1R signal was shown to be involved in Nanog-mediated self-renewal of hepatic CSCs [39]. These reports also support the importance of IGF-1R in CSC biology. In breast cancer, activation of the IGF-1R could result in stimulation of proliferation and metastasis through activation of insulin receptor substrate-1 [40] and insulin receptor substrate-2 [41]. Furthermore, it has been reported that IGF-1R expression was positively correlated with a shorter disease-free survival in triple negative breast cancer [42], the particular subtype with the highest rate of recurrence and higher percentage of BCSCs than other breast cancer subtypes [43]. In a recent report by Jones and colleagues, recurrence of breast cancer was observed in 16% of inducible IGF-1R transgenic mice upon the discontinuation of doxycycline and the recurrence involved IGF-1R-reactivation and IGF-1R-independent mechanisms [44]. Although the IGF-1R-independent tumors displayed EMT phenotypes, their metastatic potential was much lower than tumors with IGF-1R reactivation [44].

Moreover, induction of EMT in immortalized human mammary epithelial cells by overexpressing EMT-related transcriptional factors, twist or snail, or treatment with transforming growth factor β1 generated CD44^+ ^BCSCs [10]. Recently, Lorenzatti and colleagues found that CCN6, a tumor inhibitory protein, could suppress the expression of EMT transcriptional factor ZEB1 in breast cancer cells through attenuation of IGF-1R signaling [26]. Along this line, we also showed that inhibition of IGF-1R signaling suppressed the cell migration ability of CD44^+ ^BCSCs through induction of E-cadherin, the adhesion molecule that blocks the EMT process, as well as suppression of other mesenchymal markers (vimentin, twist and N-cadherin). More importantly, IGF-1R could serve as a novel marker for a particular population of cancer cells with stem/progenitor features within breast cancer since IGF-1R high-expressing human breast cancer cells displayed the capacity for mammosphere formation *in vitro *and tumorigenicity *in vivo*. Furthermore, mammosphere formation of IGF-1R expressing BCSCs was sensitive to PPP treatment. When comparing the CSC frequency of different populations of breast cancer cells, high expression of IGF-1R seems to be most efficient in enriching BCSCs in a triple negative breast cancer BC0244 xenograft (one out of 909 cells; Figure 1D). For BC0145 xenograft, the identification of cancer stem/progenitors based on IGF-1R expression (one out of 54,785; Figure 1D) was slightly better than ALDH^+ ^(one out of 93,412; see Table S1 in Additional file 1) but not as good as CD24^-^CD44^+ ^(one out of 6,401; see Table S1 in Additional file 1). These results are consistent with the known heterogeneity in the BCSC-enriched population. It has been shown that overexpression of IGF-1R in MCF7 increased the size of colonies under three-dimensional culture conditions [45], which corroborated our observation. To the best of our knowledge, this is the first demonstration of IGF-1R as a marker for cancer stem/progenitors in breast cancer.

To further investigate the downstream signaling of IGF-1R, we explored the involvement of the PI3K/Akt/mTOR pathway. The upstream stimuli of the PI3K/Akt/mTOR pathway in mammary stem/progenitor cells have been linked to Wnt/β-catenin signaling. Korkaya and colleagues demonstrated that phosphatase and tensin homolog knockdown increased phosphorylation of Akt, leading to enriched normal and malignant mammary stem/progenitor cells, and that this Akt-driven process was mediated by the Wnt/β-catenin pathway [46]. They also demonstrated that perifosine, an Akt inhibitor, could suppress both *in vivo *tumorigenicity and the *in vitro *ALDH^+ ^population of a breast cancer cell line and two xenografts of primary breast cancer [46]. In this study, we documented enhanced activation of PI3K/Akt/mTOR in BCSCs of primary human breast cancer and two xenografts of primary tumors, and suppressive effects of the mTOR inhibitor, rapamycin, on their growth *in vitro *and *in vivo*, as well as mammosphere formation. Herein, we have provided evidence for another mechanism of activation of the PI3K/Akt/mTOR pathway via IGF-1R signaling in BCSCs. The importance of PI3K/Akt/mTOR in BCSCs of clinical samples is consistent with findings from previously reported studies of breast cancer cell lines [46,47]. Enhanced phosphorylation of Akt^Ser473 ^as determined by immunohistochemical staining was reported to correlate with a poor prognosis for breast cancer [14]. In the present study, it was demonstrated for the first time that pAkt^Ser473 ^was higher in CD45^-^CD24^-^CD44^+ ^BCSCs than the non-BCSCs, in a majority (7/11, 63.6%) of those primary tumors with detectable pAkt and their xenografts in mice. There was no obvious correlation with clinical and histopathological features (see Table S2 in Additional file 1). Although BCSCs are frequently enriched in CD24^-^CD44^+ ^cells, such markers cannot be generalized to all patients, given the inherent heterogeneity of breast cancer. The variation of markers useful for enrichment of BCSCs among patients may thus explain the lack of consistent elevation of pAkt^Ser473 ^in CD24^-^CD44^+ ^cells in some patients.

Taken together, our findings support the notion that IGF-1R signaling with activation of the downstream PI3K/Akt/mTOR pathway plays an important role in breast cancer progression by controlling both the maintenance of BCSCs and their EMT behavior. These studies also provide an impetus for developing cancer therapy targeting BCSCs by combining inhibitors or mAb against IGF-1R with inhibitors of the PI3K/Akt/mTOR pathway. Although preclinical evidence for the efficacy of several small molecule inhibitors and monoclonal anti-IGF-1R antibodies (figitumumab, robatumumab and R1507) was strong, large-scale clinical trials were halted due to very modest activity [48]. The failure may be attributed in part to the selection of appropriate target population, and in part to the increased dependency of cancer cells on insulin [49]. Along this line, targeting IGF-1R and IR simultaneously with OSI-906 and BMS 754807, which are small molecule inhibitors of tyrosine kinase activity of both IGF-1R and IR, is undergoing clinical trials [48]. In addition, recent preclinical studies have shown that dual inhibition of mTOR with rapamycin and Akt with perifosine prevents mTOR inhibition-initiated Akt activation and significantly enhances antitumor effects in lung cancer [50] and multiple myeloma [51]. Also, combination of IGF-1R and mTOR inhibition showed clinical benefits in Ewing's sarcoma [52]. With a similar strategy, dalotuzumab will be combined with Akt or mTORC1 inhibition [48]. Thus, co-targeting the PI3K/Akt/mTOR pathway and its upstream signal, IGF-1R may prove to have synergistic anti-tumor effects and is worthy of further investigation.

## Conclusion

A new paradigm is emerging in cancer therapy by targeting CSCs. In this study, we demonstrated that IGF-1R could serve as a novel marker for a particular stem/progenitor population within breast cancer and its signaling pathway is critical for the survival and maintenance of BCSCs. IGF-1R silencing or small molecule inhibitors of IGF-1R and its downstream components diminished the mammosphere forming capacity and *in vivo *tumorigenicity of BCSCs. Analysis of clinical specimens of breast cancer revealed significant upregulation of phosphorylated Akt in BCSCs, which further supported the importance of this pathway. Our findings suggest that IGF-1R and its signaling via PI3K/Akt/mTOR pathway are attractive targets for therapy directed against breast cancer stem/progenitors.

## Abbreviations

ALDH: aldehyde dehydrogenase; BCSC: breast cancer stem cell; BSA: bovine serum albumin; CSC: cancer stem cell; EMT: epithelial-mesenchymal transition; ER: estrogen receptor; FACS: fluorescence-activated cell sorting; IGF-1: insulin-like growth factor-1; IGF-1R: insulin-like growth factor 1 receptor; IR: insulin receptor; mAb: monoclonal antibody; MEM: modified Eagle's medium; mTOR: mammalian target of rapamycin; PBS: phosphate-buffered saline; PI3K: phosphoinositide-3-kinase; PR: progesterone receptor; PPP: picropodophyllin; shRNA: short hairpin RNA; siRNA: small interfering RNA.

## Competing interests

The authors declare that they have no competing interests.

## Authors' contributions

W-WC designed and performed the research, analyzed data and wrote the manuscript. R-JL performed the research and analyzed the data. W-YC helped the sorting and animal experiments, C-HF helped the FACS analysis of clinical specimens. AC-YL helped the western blot analysis of IGF-1R/Akt expression. J-CY contributed clinical specimens/analytic tools. JY contributed the manuscript preparation and revision. ALY participated in the research design, data analysis and manuscript preparation. All authors read and approved the final manuscript.

## Supplementary Material

Additional file 1 **Supporting information**. Supporting information includes the methods of quantitative RT-PCR and the comparisons of histology/markers between original patient's tumor sections and xenograft tumor sections of BC0244. It also includes the validation data of BCSC characteristics of two xenograft breast cancer cells or IGF-1R+ sorted breast cancer cells. The clinical-histopathological characteristics of breast cancer patients enrolled for detection of pAkt^ser473 ^were also included here. Click here for file

## References

[B1] Nowell PC The clonal evolution of tumor cell populations Science 1976 194 23 28 10.1126/science.959840 959840

[B2] Reya T Morrison SJ Clarke MF Weissman IL Stem cells, cancer, and cancer stem cells Nature 2001 414 105 111 10.1038/35102167 11689955

[B3] Lapidot T Sirard C Vormoor J Murdoch B Hoang T Caceres-Cortes J Minden M Paterson B Caligiuri MA Dick JE A cell initiating human acute myeloid leukaemia after transplantation into SCID mice Nature 1994 367 645 648 10.1038/367645a0 7509044

[B4] Singh SK Hawkins C Clarke ID Squire JA Bayani J Hide T Henkelman RM Cusimano MD Dirks PB Identification of human brain tumour initiating cells Nature 2004 432 396 401 10.1038/nature03128 15549107

[B5] Al-Hajj M Wicha MS Benito-Hernandez A Morrison SJ Clarke MF Prospective identification of tumorigenic breast cancer cells Proc Natl Acad Sci USA 2003 100 3983 3988 10.1073/pnas.0530291100 12629218 PMC153034

[B6] O'Brien CA Pollett A Gallinger S Dick JE A human colon cancer cell capable of initiating tumour growth in immunodeficient mice Nature 2007 445 106 110 10.1038/nature05372 17122772

[B7] Ginestier C Hur MH Charafe-Jauffret E Monville F Dutcher J Brown M Jacquemier J Viens P Kleer CG Liu S Schott A Hayes D Birnbaum D Wicha MS Dontu G ALDH1 is a marker of normal and malignant human mammary stem cells and a predictor of poor clinical outcome Cell Stem Cell 2007 1 555 567 10.1016/j.stem.2007.08.014 18371393 PMC2423808

[B8] Phillips TM McBride WH Pajonk F The response of CD24^-/low^/CD44^+ ^breast cancer-initiating cells to radiation J Natl Cancer Inst 2006 98 1777 1785 10.1093/jnci/djj495 17179479

[B9] Balic M Lin H Young L Hawes D Giuliano A McNamara G Datar RH Cote RJ Most early disseminated cancer cells detected in bone marrow of breast cancer patients have a putative breast cancer stem cell phenotype Clin Cancer Res 2006 12 5615 5621 10.1158/1078-0432.CCR-06-0169 17020963

[B10] Mani SA Guo W Liao MJ Eaton EN Ayyanan A Zhou AY Brooks M Reinhard F Zhang CC Shipitsin M Campbell LL Polyak K Brisken C Yang J Weinberg RA The epithelial-mesenchymal transition generates cells with properties of stem cells Cell 2008 133 704 715 10.1016/j.cell.2008.03.027 18485877 PMC2728032

[B11] Charafe-Jauffret E Monville F Ginestier C Dontu G Birnbaum D Wicha MS Cancer stem cells in breast: current opinion and future challenges Pathobiology 2008 75 75 84 10.1159/000123845 18544962 PMC2789397

[B12] Blume-Jensen P Hunter T Oncogenic kinase signalling Nature 2001 411 355 365 10.1038/35077225 11357143

[B13] Bose S Chandran S Mirocha JM Bose N The Akt pathway in human breast cancer: a tissue-array-based analysis Modern Pathol 2006 19 238 245 10.1038/modpathol.3800525 16341149

[B14] Perez-Tenorio G Stal O Activation of AKT/PKB in breast cancer predicts a worse outcome among endocrine treated patients Br J Cancer 2002 86 540 545 10.1038/sj.bjc.6600126 11870534 PMC2375266

[B15] Jiang BH Liu LZ PI3K/PTEN signaling in tumorigenesis and angiogenesis Biochim Biophys Acta 2008 1784 150 158 10.1016/j.bbapap.2007.09.008 17964232

[B16] Pollak M Insulin and insulin-like growth factor signalling in neoplasia Nat Rev Cancer 2008 8 915 928 10.1038/nrc2536 19029956

[B17] Paik S Expression of IGF-I and IGF-II mRNA in breast tissue Breast Cancer Res Treat 1992 22 31 38 10.1007/BF01833331 1421422

[B18] Furstenberger G Senn HJ Insulin-like growth factors and cancer Lancet Oncol 2002 3 298 302 10.1016/S1470-2045(02)00731-3 12067807

[B19] Surmacz E Function of the IGF-I receptor in breast cancer J Mammary Gland Biol Neoplasia 2000 5 95 105 10.1023/A:1009523501499 10791772

[B20] Chang WW Lee CH Lee P Lin J Hsu CW Hung JT Lin JJ Yu JC Shao LE Yu J Wong CH Yu AL Expression of Globo H and SSEA3 in breast cancer stem cells and the involvement of fucosyl transferases 1 and 2 in Globo H synthesis Proc Natl Acad Sci USA 2008 105 11667 11672 10.1073/pnas.0804979105 18685093 PMC2575305

[B21] Hu Y Smyth GK ELDA: extreme limiting dilution analysis for comparing depleted and enriched populations in stem cell and other assays J Immunol Methods 2009 347 70 78 10.1016/j.jim.2009.06.008 19567251

[B22] Fulzele K DiGirolamo DJ Liu Z Xu J Messina JL Clemens TL Disruption of the insulin-like growth factor type 1 receptor in osteoblasts enhances insulin signaling and action J Biol Chem 2007 282 25649 25658 10.1074/jbc.M700651200 17553792

[B23] Girnita A Girnita L del Prete F Bartolazzi A Larsson O Axelson M Cyclolignans as inhibitors of the insulin-like growth factor-1 receptor and malignant cell growth Cancer Res 2004 64 236 242 10.1158/0008-5472.CAN-03-2522 14729630

[B24] Saxena NK Taliaferro-Smith L Knight BB Merlin D Anania FA O'Regan RM Sharma D Bidirectional crosstalk between leptin and insulin-like growth factor-I signaling promotes invasion and migration of breast cancer cells via transactivation of epidermal growth factor receptor Cancer Res 2008 68 9712 9722 10.1158/0008-5472.CAN-08-1952 19047149 PMC3180854

[B25] Sachdev D Regulation of breast cancer metastasis by IGF signaling J Mammary Gland Biol Neoplasia 2008 13 431 441 10.1007/s10911-008-9105-5 19030970

[B26] Lorenzatti G Huang W Pal A Cabanillas AM Kleer CG CCN6 (WISP3) decreases ZEB1-mediated EMT and invasion by attenuation of IGF-1 receptor signaling in breast cancer J Cell Sci 2011 124 1752 1758 10.1242/jcs.084194 21525039 PMC3085438

[B27] Calcagno AM Salcido CD Gillet JP Wu CP Fostel JM Mumau MD Gottesman MM Varticovski L Ambudkar SV Prolonged drug selection of breast cancer cells and enrichment of cancer stem cell characteristics J Natl Cancer Inst 2010 102 1637 1652 10.1093/jnci/djq361 20935265 PMC2970576

[B28] To K Fotovati A Reipas KM Law JH Hu K Wang J Astanehe A Davies AH Lee L Stratford AL Raouf A Johnson P Berquin IM Royer HD Eaves CJ Dunn SE Y-box binding protein-1 induces the expression of CD44 and CD49f leading to enhanced self-renewal, mammosphere growth, and drug resistance Cancer Res 2010 70 2840 2851 10.1158/0008-5472.CAN-09-3155 20332234 PMC2848879

[B29] Blows FM Driver KE Schmidt MK Broeks A van Leeuwen FE Wesseling J Cheang MC Gelmon K Nielsen TO Blomqvist C Heikkila P Heikkinen T Nevanlinna H Akslen LA Begin LR Foulkes WD Couch FJ Wang X Cafourek V Olson JE Baglietto L Giles GG Severi G McLean CA Southey MC Rakha E Green AR Ellis IO Sherman ME Lissowska J Subtyping of breast cancer by immunohistochemistry to investigate a relationship between subtype and short and long term survival: a collaborative analysis of data for 10,159 cases from 12 studies PLoS Med 2010 7 e1000279 10.1371/journal.pmed.1000279 20520800 PMC2876119

[B30] Onitilo AA Engel JM Greenlee RT Mukesh BN Breast cancer subtypes based on ER/PR and Her2 expression: comparison of clinicopathologic features and survival Clin Med Res 2009 7 4 13 10.3121/cmr.2008.825 19574486 PMC2705275

[B31] Vanamala J Reddivari L Radhakrishnan S Tarver C Resveratrol suppresses IGF-1 induced human colon cancer cell proliferation and elevates apoptosis via suppression of IGF-1R/Wnt and activation of p53 signaling pathways BMC Cancer 2010 10 238 10.1186/1471-2407-10-238 20504360 PMC2891636

[B32] Ye P Hu Q Liu H Yan Y D'Ercole AJ beta-catenin mediates insulin-like growth factor-I actions to promote cyclin D1 mRNA expression, cell proliferation and survival in oligodendroglial cultures Glia 2010 58 1031 1041 10.1002/glia.20984 20235220 PMC2917840

[B33] Wang L Shao YY Ballock RT Thyroid hormone-mediated growth and differentiation of growth plate chondrocytes involves IGF-1 modulation of beta-catenin signaling J Bone Mineral Res 2010 25 1138 1146 10.1002/jbmr.5 PMC3123724 20200966

[B34] Medyouf H Gusscott S Wang H Tseng JC Wai C Nemirovsky O Trumpp A Pflumio F Carboni J Gottardis M Pollak M Kung AL Aster JC Holzenberger M Weng AP High-level IGF1R expression is required for leukemia-initiating cell activity in T-ALL and is supported by Notch signaling J Exp Med 2011 208 1809 1822 10.1084/jem.20110121 21807868 PMC3171095

[B35] Jenkins CR Shevchuk OO Giambra V Lam SH Carboni JM Gottardis MM Holzenberger M Pollak M Humphries RK Weng AP IGF signaling contributes to malignant transformation of hematopoietic progenitors by the MLL-AF9 oncoprotein Exp Hematol 2012 40 715 723 e6 10.1016/j.exphem.2012.05.003 22613471

[B36] Grudzien P Lo S Albain KS Robinson P Rajan P Strack PR Golde TE Miele L Foreman KE Inhibition of Notch signaling reduces the stem-like population of breast cancer cells and prevents mammosphere formation Anticancer Res 2010 30 3853 3867 21036696

[B37] Farnie G Clarke RB Mammary stem cells and breast cancer - role of Notch signalling Stem Cell Rev 2007 3 169 175 10.1007/s12015-007-0023-5 17873349

[B38] Dallas NA Xia L Fan F Gray MJ Gaur P van Buren G Samuel S Kim MP Lim SJ Ellis LM Chemoresistant colorectal cancer cells, the cancer stem cell phenotype, and increased sensitivity to insulin-like growth factor-I receptor inhibition Cancer Res 2009 69 1951 1957 10.1158/0008-5472.CAN-08-2023 19244128 PMC3198868

[B39] Shan J Shen J Liu L Xia F Xu C Duan G Xu Y Ma Q Yang Z Zhang Q Ma L Liu J Xu S Yan X Bie P Cui Y Bian XW Qian C Nanog regulates self-renewal of cancer stem cells through the insulin-like growth factor pathway in human hepatocellular carcinoma Hepatology 2012 56 1004 1014 10.1002/hep.25745 22473773

[B40] Jackson JG White MF Yee D Insulin receptor substrate-1 is the predominant signaling molecule activated by insulin-like growth factor-I, insulin, and interleukin-4 in estrogen receptor-positive human breast cancer cells J Biol Chem 1998 273 9994 10003 10.1074/jbc.273.16.9994 9545345

[B41] Jackson JG Zhang X Yoneda T Yee D Regulation of breast cancer cell motility by insulin receptor substrate-2 (IRS-2) in metastatic variants of human breast cancer cell lines Oncogene 2001 20 7318 7325 10.1038/sj.onc.1204920 11704861

[B42] Hartog H Horlings HM van der Vegt B Kreike B Ajouaou A van de Vijver MJ Marike Boezen H de Bock GH van der Graaf WT Wesseling J Divergent effects of insulin-like growth factor-1 receptor expression on prognosis of estrogen receptor positive versus triple negative invasive ductal breast carcinoma Breast Cancer Res Treat 2010 129 725 736 21107683 10.1007/s10549-010-1256-6

[B43] Stratford AL Reipas K Maxwell C Dunn SE Targeting tumour-initiating cells to improve the cure rates for triple-negative breast cancer Expert Rev Mol Med 2010 12 e22 20653987 10.1017/S1462399410001535

[B44] Jones RA Campbell CI Wood GA Petrik JJ Moorehead RA Reversibility and recurrence of IGF-IR-induced mammary tumors Oncogene 2009 28 2152 2162 10.1038/onc.2009.79 19377512

[B45] Zhang Y Moerkens M Ramaiahgari S de Bont H Price L Meerman J van de Water B Elevated insulin-like growth factor 1 receptor signaling induces antiestrogen resistance through the MAPK/ERK and PI3K/Akt signaling routes Breast Cancer Res 2011 13 R52 10.1186/bcr2883 21595894 PMC3218939

[B46] Korkaya H Paulson A Charafe-Jauffret E Ginestier C Brown M Dutcher J Clouthier SG Wicha MS Regulation of mammary stem/progenitor cells by PTEN/Akt/beta-catenin signaling PLoS Biol 2009 7 e1000121 10.1371/journal.pbio.1000121 19492080 PMC2683567

[B47] Zhou J Wulfkuhle J Zhang H Gu P Yang Y Deng J Margolick JB Liotta LA Petricoin E Zhang Y Activation of the PTEN/mTOR/STAT3 pathway in breast cancer stem-like cells is required for viability and maintenance Proc Natl Acad Sci USA 2007 104 16158 16163 10.1073/pnas.0702596104 17911267 PMC2042178

[B48] Yee D Insulin-like growth factor receptor inhibitors: baby or the bathwater? J Natl Cancer Inst 2012 104 975 981 10.1093/jnci/djs258 22761272 PMC3634550

[B49] Zhang H Pelzer AM Kiang DT Yee D Down-regulation of type I insulin-like growth factor receptor increases sensitivity of breast cancer cells to insulin Cancer Res 2007 67 391 397 10.1158/0008-5472.CAN-06-1712 17210722

[B50] Wang X Yue P Kim YA Fu H Khuri FR Sun SY Enhancing mammalian target of rapamycin (mTOR)-targeted cancer therapy by preventing mTOR/raptor inhibition-initiated, mTOR/rictor-independent Akt activation Cancer Res 2008 68 7409 7418 10.1158/0008-5472.CAN-08-1522 18794129 PMC2562339

[B51] Cirstea D Hideshima T Rodig S Santo L Pozzi S Vallet S Ikeda H Perrone G Gorgun G Patel K Desai N Sportelli P Kapoor S Vali S Mukherjee S Munshi NC Anderson KC Raje N Dual inhibition of akt/mammalian target of rapamycin pathway by nanoparticle albumin-bound-rapamycin and perifosine induces antitumor activity in multiple myeloma Mol Cancer Ther 2010 9 963 975 10.1158/1535-7163.MCT-09-0763 20371718 PMC3096071

[B52] Naing A LoRusso P Fu S Hong DS Anderson P Benjamin RS Ludwig J Chen HX Doyle LA Kurzrock R Insulin growth factor-receptor (IGF-1R) antibody cixutumumab combined with the mTOR inhibitor temsirolimus in patients with refractory Ewing's sarcoma family tumors Clin Cancer Res 2012 18 2625 2631 10.1158/1078-0432.CCR-12-0061 22465830 PMC3875297

[B53] Munster PN Srethapakdi M Moasser MM Rosen N Inhibition of heat shock protein 90 function by ansamycins causes the morphological and functional differentiation of breast cancer cells Cancer Res 2001 61 2945 2952 11306472

