# Supplementary material for: The expression and significance of insulin-like growth factor-1 receptor and its pathway on breast cancer stem/progenitors
Source: Breast Cancer Res. 2013 May 12;15(3):R39. doi: 10.1186/bcr3423 (PMC3706809; doi:10.1186/bcr3423)
Supplement: Additional file 1 — Supporting information. Supporting information includes the methods of quantitative RT-PCR and the comparisons of histology/markers between original patient's tumor sections and xenograft tumor sections of BC0244. It also includes the validation data of BCSC characteristics of two xenograft breast cancer cells or IGF-1R+ sorted breast cancer cells. The clinical-histopathological characteristics of breast cancer patients enrolled for detection of pAktser473 were also included here. [file bcr3423-S1.PDF]

Supporting information

Materials and Methods

Quantitative RT-PCR

The mRNA level of *IGR-1R*, *Akt*, *mTOR* and *GAPD*H were determined by SYBR Green based quantitative RT-PCR. 10ng of cellular cDNA and specific primer pairs (final concentration as 50nM) was mixed with Power SYBR® Green Master mix (Applied Biosystems) and fluorescence signal was detected by ABI Prism 7000 Sequence Detection System (Applied Biosystems). The Ct values were calculated by ABI software. Expression level of *IGF-1R*, *Akt* or *mTOR* in BCSCs or nonBCSCs was normalized by *GAPDH* (△Ct). The differential expression of IGF-1R, Akt, mTOR between BCSCs and nonBCSCs was calculated by $2^{-(\triangle Ct(BCSCs)-\triangle Ct(nonBCSC))}$.The primer sets used were listed below:

Akt1-forward: 5'-TCCCGAGGCCAAGTCCTT-3'
Akt1-reverse: 5'-CCGCCAAGCCTCTGCTT-3'
mTOR-forward: 5'-AGCATCGGATGCTTAGGAGTGG-3'
*mTO*R-reverse: 5'- CAGCCAGTCATCTTTGGAGACC-3'
IGF-1R-forward: 5'- CCTGCACAACTCCATCTTCGTG-3'
IGF-1R-reverse: 5'- CGGTGATGTTGTAGGTGTCTGC-3'
GAPDH-forward: 5'-CGGGAAACTGTGGCGTGATG-3'
GAPDH-reverse: 5'-TGGAGGAGTGGGTGTCGCTGTT-3'

Figures

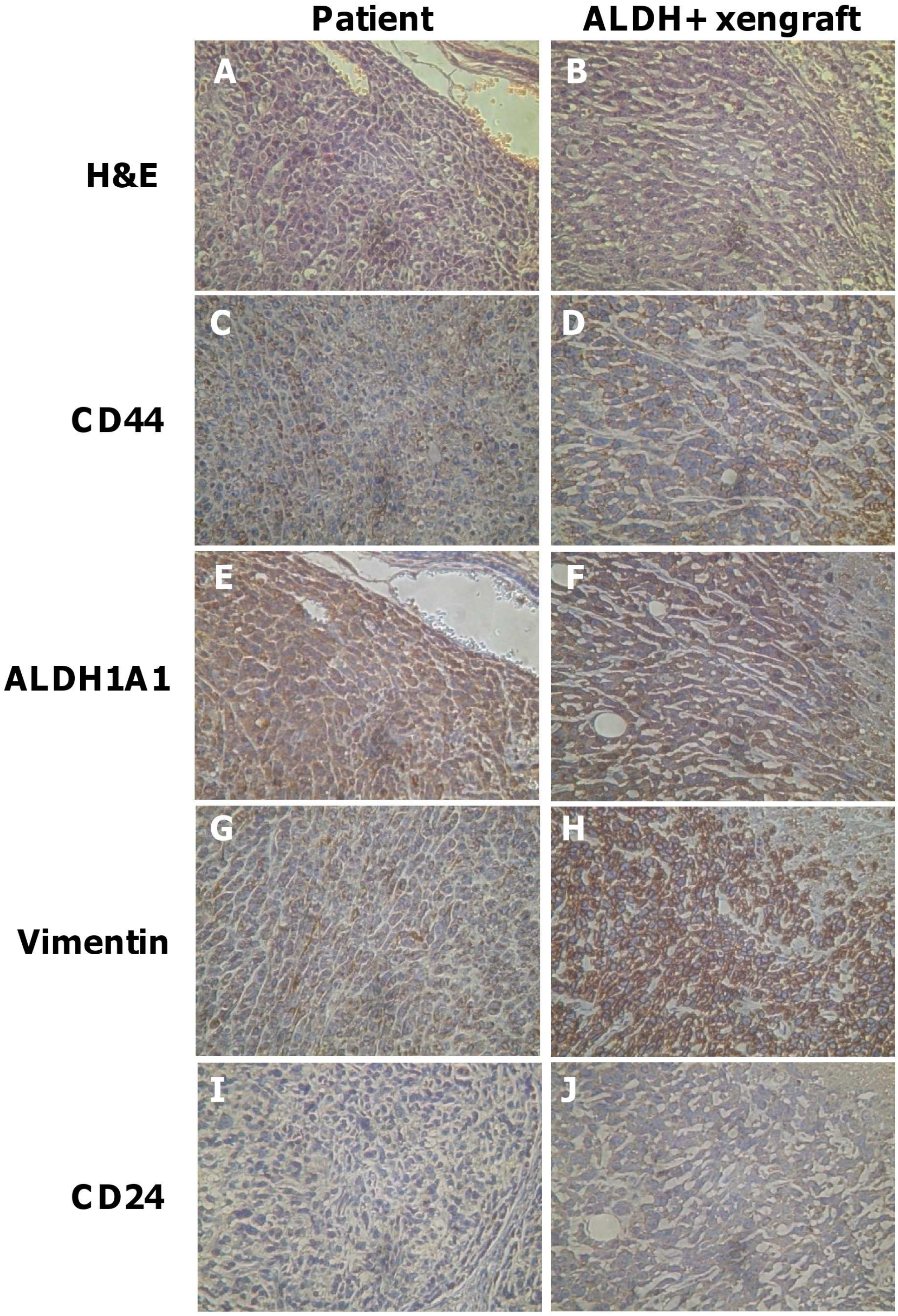

Figure S1. Histological and immunohistochemistrical comparison between BC0244 xenograft tumor and its parental patient's tumor. The paraffin sections of BC0244 xenograft tumor and its parental patient's tumor were stained with H&E (A, B), ALDH1A1(C, D), CD44 (E, F), vimentin (G, H) or CD24 (I, J). Red color indicated positive signal in C to J.

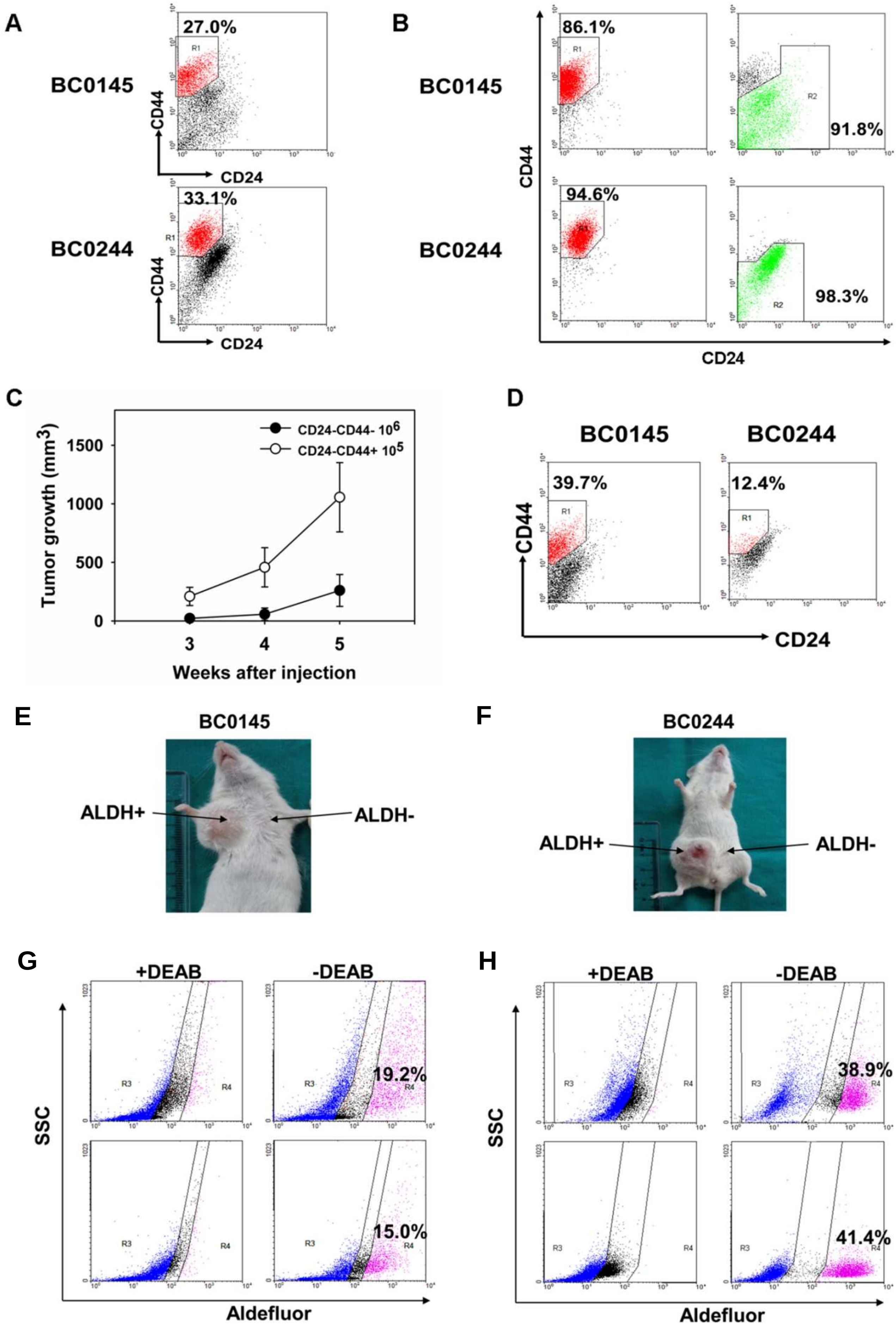
A
BC0145
27.0%
CD44
CD24
33.1%
BC0244
B
86.1%
91.8%
94.6%
98.3%
CD44
CD24
C
Tumor growth (mm3)
CD24-CD44- 10^6
CD24-CD44+ 10^5
Weeks after injection
D
BC0145
BC0244
39.7%
12.4%
CD44
CD24
E
BC0145
ALDH+
ALDH-
F
BC0244
ALDH+
ALDH-
G
+DEAB
-DEAB
SSC
19.2%
15.0%
Aldefluor
H
+DEAB
-DEAB
SSC
38.9%
41.4%
Aldefluor

Figure S2. Enrichment of breast cancer stem cell (BCSC) populations based on $CD24^{-}CD44^{+}$ or ALDH markers in two xenografts of human primary breast cancer. (A) Phenotypic analysis of BC0145 and BC0244 xenografts by surface markers CD24 and CD44. (B) The purities of $H2K^{d-}CD24^{-}CD44^{+}$ and $H2K^{d-}CD24^{-}CD44^{-}$ cells sorted from BC0145 and BC0244 xenografts. The purities were 86.1% and 91.8% for BC0145, and 94.6% and 98.3% for BC0244, respectively. The sorted cells were injected into mammary fat pads of NOD/SCID mice, and the results of tumor formation are shown in Table 1. (C) The tumor volume after injecting the indicated number of two populations of cells sorted from BC0145, as determined by the formula: $D \times d^2 \times \pi/6$ (*D*, longer diameter; *d*, shorter diameter). (D) Phenotypic diversity in tumors engrafted from $CD24^{-}CD44^{+}$ cells of BC0145 and BC0244 as determined by flow cytometry. (E-F) Cells were isolated from BC0145 and BC0244 xenograft tumors and their ALDH activities were determined by an Aldefluor assay. Different numbers of sorted cells with high ($ALDH^{+}$) and low ALDH activity ($ALDH^{-}$) were injected into mammary fat pads of NOD/SCID mice. Tumorigenicity results are summarized in Table 1. Pictures of mice were taken on days 72 (E, BC0145) and 58 (F, BC0244). (G-H) The phenotypic diversities of $ALDH^{+}$- tumors derived from BC0145 (G) and BC0244 (H) were examined by the Aldefluor assay in the presence or absence of ALDH1 inhibitor, DEAB. Upper panel represented the results of original tumor cells subjected to sorting and lower panel showed the data of tumor cells derived from xenograft of $ALDH^{+}$ cells.

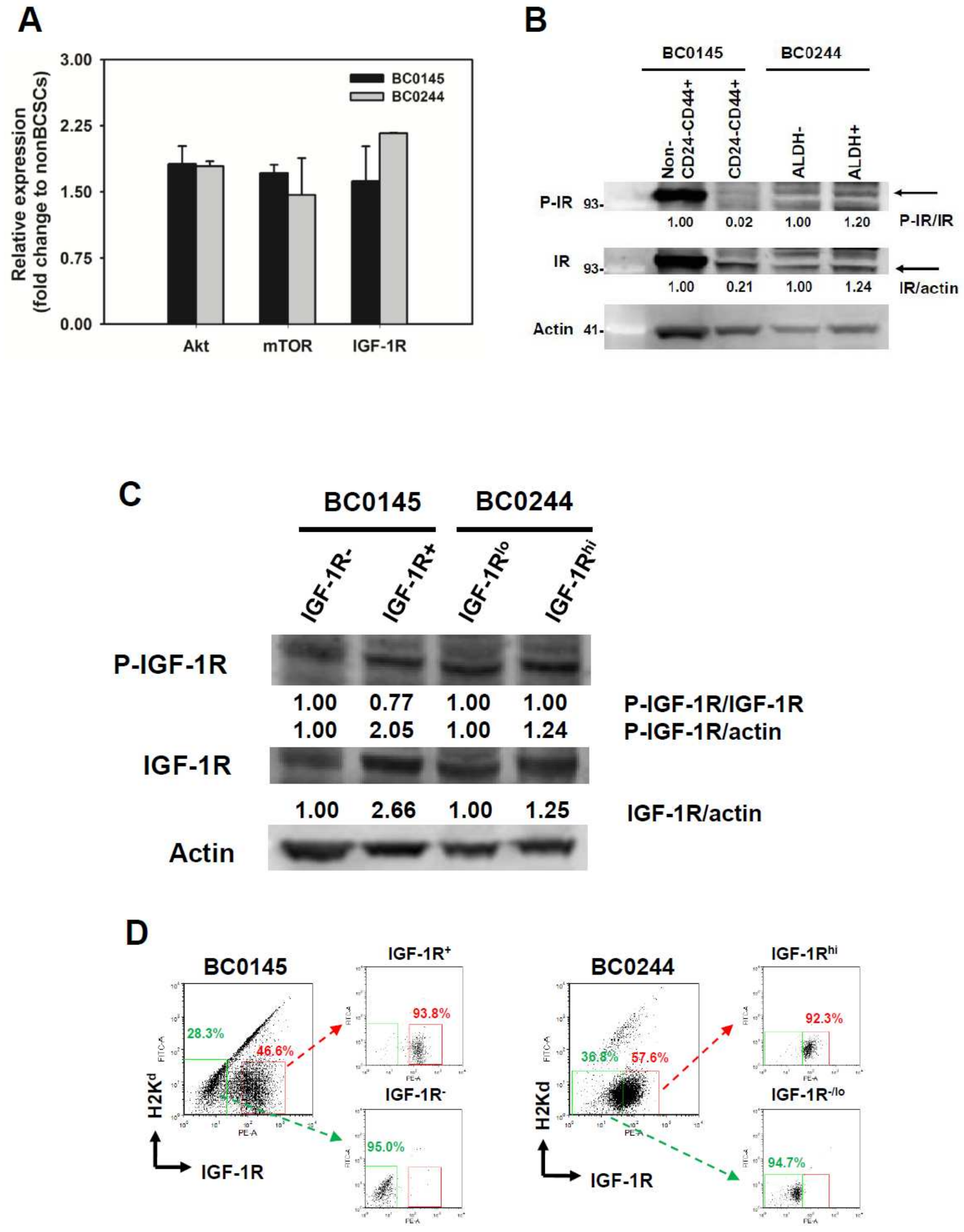

Figure S3. Comparison of mRNA levels of *IGF-1R/Akt/mTOR* or p-IR/IR in BCSCs and non-BCSCs and IGF-1R expression in IGF-1R+ sorted xenografted tumor cells. (A) 10ng of cDNA from BCSCs ($CD24^{-}CD44^{+}$ for BC0145 and $ALDH^{+}$ for BC0244) or nonBCSCs ($CD24^{-}CD44^{-}$ for BC0145 and $ALDH^{-}$ for BC0244) were used for determination

of the expression of *IGF-1R*, *Akt*, *mTOR* or *GAPDH* by SYBR Green based quantitative RT-PCR. The relative expressions of *IGF-1R*, *Akt* or *mTOR* in BCSCs to non-BCSCs were calculated by formula described in "Materials and Methods" section of Supporting Information. (B) BCSCs and non-BCSCs were sorted from BC0145 or BC0244 xenografted tumor cells and the expression of p-IR and IR was determined by western blot. Actin was used as internal control. (C) BC0145 and BC0244 xenografted tumor cells were sorted into IGF-1R$^-$ and IGF-1R$^+$, or IGF-1R^{lo} and IGF-1R^{hi} cells, respectively by cell sorter and their cell lysates were analyzed for phospho- IGF-1R and total IGF-1R by western blot. Actin was used as internal control. (D) BC0145 or BC0244 xenografted tumor cells were stained with anti-H2K^d-FITC and anti-IGF-1R-PE antibodies and performed cell sorting as described "Materials and Methods" section. The purity of sorted cells was confirmed by FACS.

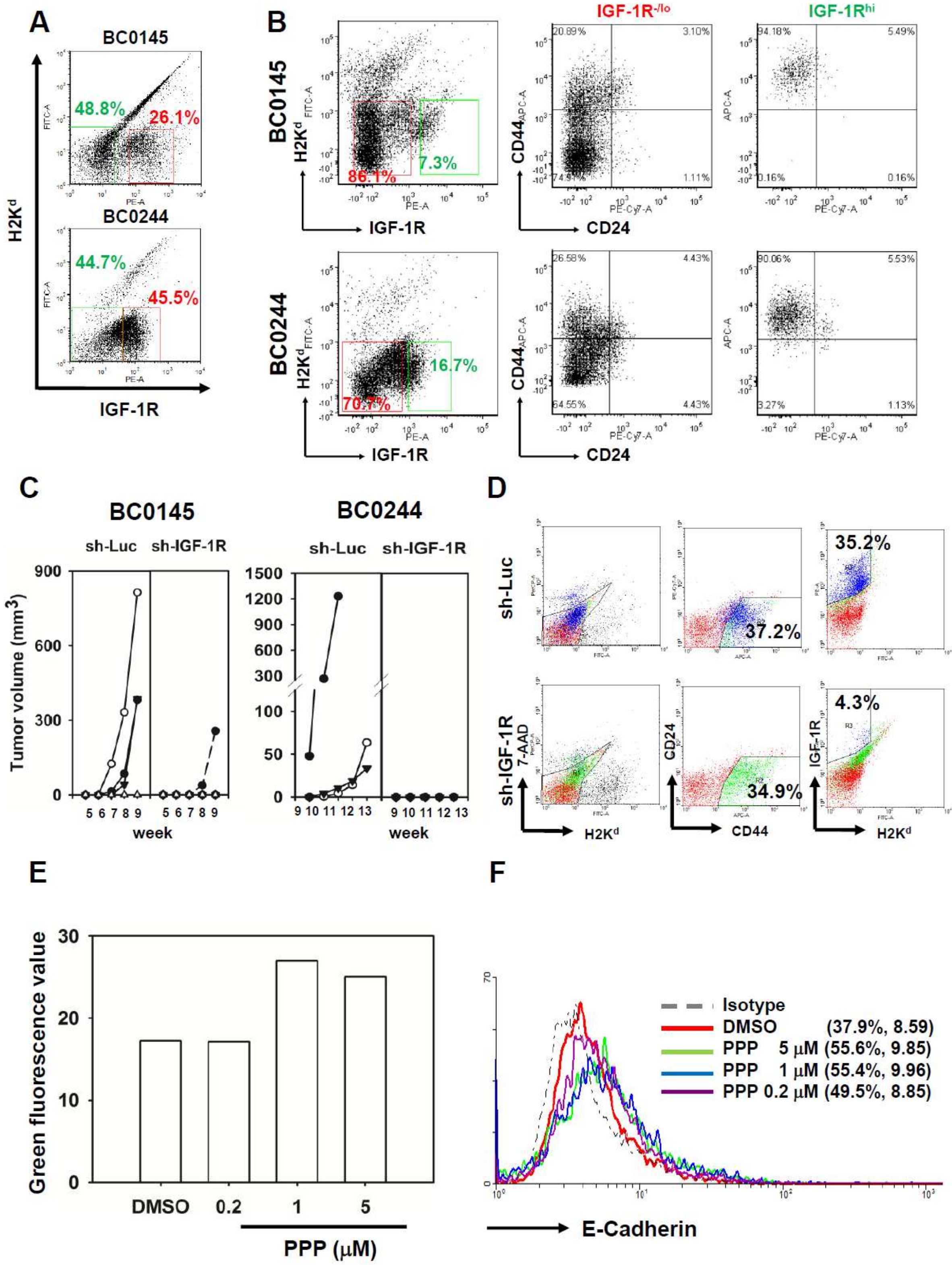

Figure S4. IGF-1R+ breast cancer cells display the properties of stem/progenitors. (A)

Tumors formed from IGF-1R$^+$ (for BC0145) or IGF-1R^{hi} (for BC0244) cells were harvested and determined the IGF-1R expression by FACS. (B) Tumors cells derived from IGF-1R$^+$ BC0145 cells or IGF-1R^{hi} BC0244 cells were stained with fluorescent-conjugated anti-IGF-1R, anti-CD24 and anti-CD44 antibodies and were further analyzed the percentage of CD24$^-$CD44$^+$ cells within IGF-1R$^{-/lo}$ or IGF-1R^{hi} cells. (C) IGF-1R$^+$ BC0145 or IGF-1R^{hi} BC0244 cells were sorted, transduced with sh-Luc or sh-IGF-1R lentivirus and the tumor growth curve was monitored weekly. (C) CD24$^-$CD44$^+$ or IGF-1R$^+$ cells within in tumors formed from sh-Luc- (upper panel) or sh-IGF-1R-(lower panel) transduced BC0145 cells were determined by FACS. (D) Green fluorescence intensity of immunofluorescent staining of E-cadherin which displayed in Figure 1E was quantified by ImageJ software. (E) E-cadherin expression of PPP-treated BCSCs was confirmed by FACS. Quantitative data were presented in parenthesis as (percentage, mean fluorescence intensity of E-cadherin expressing cells)

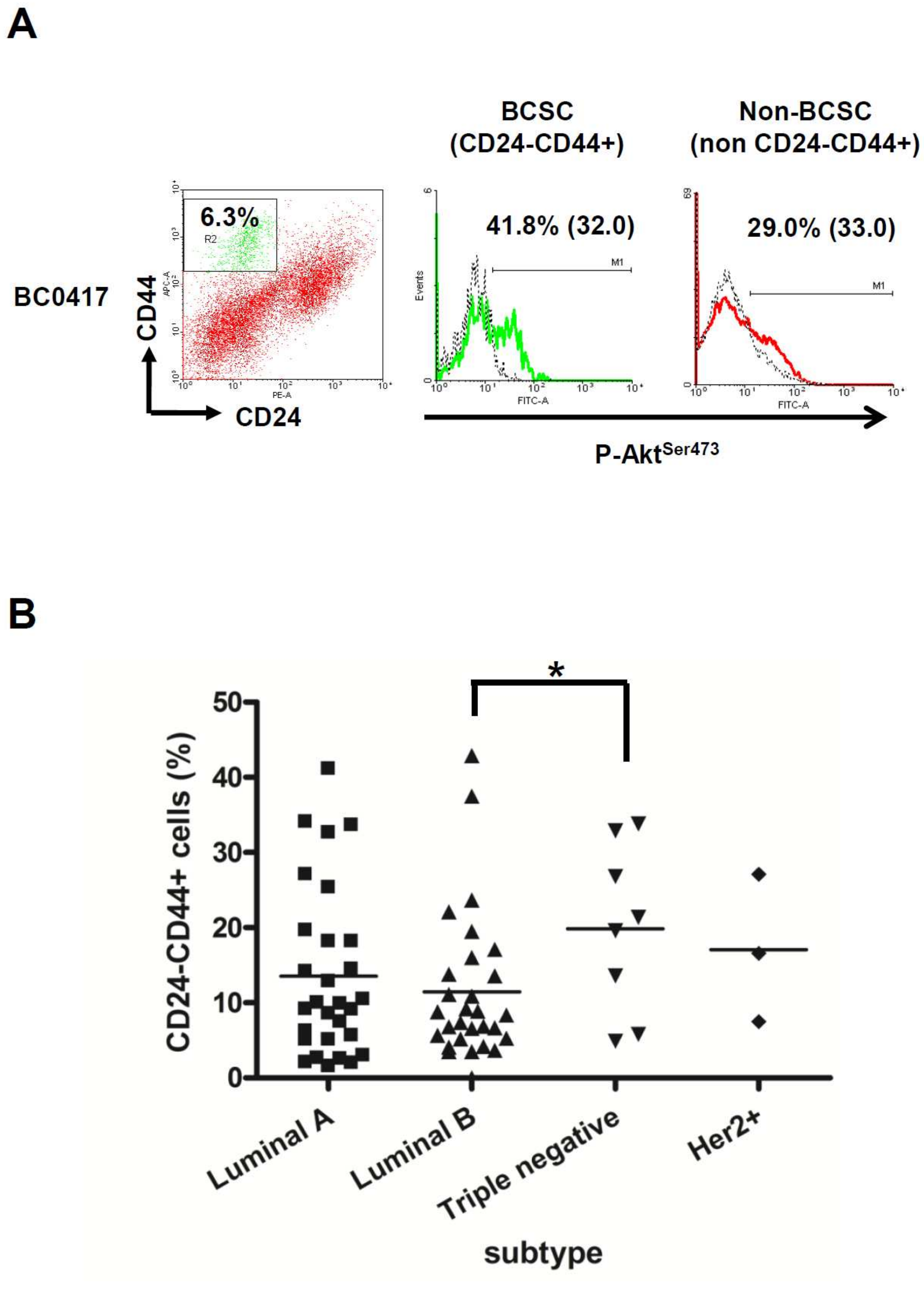

Figure S5. FACS analysis of p-Akt^{ser473} expression in breast cancer specimens and the correlation of CD24$^-$CD44$^+$ with breast cancer subtypes. (A) BCSCs were gated as CD45$^-$CD24$^-$CD44$^+$ and the remaining cells were considered as non-BCSCs. Dotted lines represented isotype control. The data were presented as percentage (mean fluorescence

intensity) of phosphor-Akt expressing cells. (B) The expression of $CD24^{-}CD44^{+}$ cells was analyzed by FACS among 52 breast cancer specimens (11 in this study and 41 from our previous report). The breast cancer subtypes were categorized according to the expression status of ER, PR and HER2/neu as follows: ER/PR+, HER2/neu- for luminal A; ER/PR+, HER2/neu+ for luminal B; ER-, PR-, HER2/neu+ for HER2+; ER-, PR-, HER2/neu- for triple negative. *, $p<0.05$.

Tables

Table S1. Engraftment of tumors in NOD/SCID mice with different cell populations isolated from BC0145 and BC0244 xenograft tumor cells.

	Injected Cell no.	Engraftment rate	Tumor weight (g)	CSC Frequency[*]	P value[*]
**BC0145**					
**$H2K^{d-}CD24^{-}CD44^{-}$**	**10^6**	**3/3**	**1.35 ± 0.48[†]**	**1:645515**	**1.28×10^{-185}**
	**5×10^5**	**0/2[‡]**	**-**		
	**10^5**	**0/3[§]**	**-**		
	**5×10^4**	**1/3**	**ND[¶]**		
	**10^4**	**0/3[§]**	**-**		
**$H2K^{d-}CD24^{-}CD44^{+}$**	**10^5**	**3/3**	**2.35 ± 0.85[†]**	**1:6041**	
	**10^4**	**4/5**	**ND[¶]**		
	**10^3**	**1/6**	**ND[¶]**		
**$H2K^{d-}ALDH^{-}$**	**10^6**	**0/5[‖]**	**-**	**1:5545027**	**1.69×10^{-74}**
	**10^5**	**0/5[‖]**	**-**		
	**10^4**	**1/5**	**ND[¶]**		
**$H2K^{d-}ALDH^{+}$**	**10^5**	**3/5**	**ND[¶]**	**1:93412**	
	**10^4**	**1/5**	**ND[¶]**		
	**10^3**	**1/5**	**ND[¶]**		
**BC0244**					
**$H2K^{d-}CD24^{-}CD44^{-}$**	**10^6**	**0/3[**]**	**-**	**1:4144998**	**2.65×10^{-118}**
	**3.5×10^5**	**0/2[††]**	**-**		
	**10^5**	**0/3[††]**	**-**		
	**5×10^4**	**0/2[††]**	**-**		
	**10^4**	**1/5**	**ND[¶]**		
**$H2K^{d-}CD24^{-}CD44^{+}$**	**10^5**	**3/5**	**ND[¶]**	**1:48710**	
	**10^4**	**3/5**	**ND[¶]**		
	**10^3**	**1/5**	**ND[¶]**		
**$H2K^{d-}ALDH^{-}$**	**2.5×10^5**	**0/5[††]**	**-**	**< 1:462000**	**3.28×10^{-166}**
	**2.5×10^4**	**0/5[††]**	**-**		
	**2.5×10^3**	**0/3[‡‡]**	**-**		
**$H2K^{d-}ALDH^{+}$**	**10^5**	**4/5**	**ND[‖]**	**1:12740**	
	**10^4**	**4/5**	**ND[‖]**		
	**10^3**	**3/5**	**ND[‖]**		

*CSC frequency and p value was calculated by Extreme Limiting Dilution Analysis (ELDA) website software.

[†]Mice were sacrificed at week 6.

[‡] Tumor free on Day 186.

[§] Tumor free at Day 91.

[¶] Not determined.

[‖] Tumor free on Day 149 to Day 280.

[**] Tumor free on Day 140.

[††] Tumor free on Day 156.

Table S2. Clinical-histopathological characteristics of breast cancer patients and the expression of pAkt^{Ser473} in their tumors.

Coding	Age	Stage	Tumor type	ER*	PR*	HER2/neu*	pAkt^{Ser473}
BC0408	61	I	Invasive ductal	+	+	+	0
C0410	40	I	Invasive ductal	+	+	-	0
BC0411	60	II	Invasive ductal	+	-	+	0
BC0414	49	II	Solid papillary	-	-	-	19.2
BC0417	59	II	Invasive ductal	-	-	-	29.7
BC0422	79	III	Invasive lobular	+	+	-	15.3
BC0426	68	II	Invasive ductal	-	-	-	22.8
BC0450	70	II	Invasive ductal	+	+	-	54.1
BC0466	48	I	Invasive ductal	+	+	-	42.9
BC0468	49	0	Ductal carcinoma in	-	-	+	0
BC0480	60	II	Invasive ductal	+	-	+	39.4

**BC0485**	**40**	**II**	**Invasive ductal**	+	**-**	**-**	**41.5**
**BC0512**	**73**	**II**	**Invasive ductal**	+	**-**	+	**39.1**
**BC0524**	**63**	**III**	**Invasive ductal**	+	+	+	**0**
**BC0526**	**47**	**II**	**Invasive lobular**	+	+	+	**51.1**
**BC0533**	**47**	**I**	**Invasive ductal**	+	+	+	**10.6**

*ER, estrogen receptor; PR, progesterone receptor. The expression of ER, PR and HER2/neu was determined by immunohistochemical staining of paraffin section.

†pAkt^{Ser473} expression was determined by FACS analysis and the percent of total cell population expressing pAkt was shown here.

Table S3. Relationship between p-Akt^{Ser473} expression and clinical- histopathological features in primary breast cancer specimens.

Histopatholoical factor	Positive rate of detectable p-Akt^{ser473} (%)	p-Akts^{er473} expression level (% of total analyzed tumor cells)	p-value*
**Stage**			**0.112**
**0**	**0**	**0**	
**I**	**50**	**0-42.9**	
**II**	**88.9**	**0-54.1**	
**III**	**50**	**0-15.3**	
**ER**			**0.585**
**+**	**66.7**	**0-54.1**	
**-**	**75.0**	**0-29.7**	
**PR**			**0.833**
**+**	**62.5**	**0-54.1**	
**-**	**75.0**	**0-41.5**	
**HER2/neu**			**0.300**
**+**	**50.0**	**0-51.1**	
**-**	**87.5**	**0-54.1**	

*p-value of Stage was calculated by one-way ANOVA and others was calculated by unpaired

t-test.
